# Analysis of sampling artifacts on the Granger causality analysis for topology extraction of neuronal dynamics

**DOI:** 10.3389/fncom.2014.00075

**Published:** 2014-07-30

**Authors:** Douglas Zhou, Yaoyu Zhang, Yanyang Xiao, David Cai

**Affiliations:** ^1^Department of Mathematics, MOE-LSC, and Institute of Natural Sciences, Shanghai Jiao Tong UniversityShanghai, China; ^2^Courant Institute of Mathematical Sciences and Center for Neural Science, New York UniversityNew York, NY, USA; ^3^NYUAD Institute, New York University Abu DhabiAbu Dhabi, UAE

**Keywords:** Granger causality, sampling rate, causal inference, reliability, neuronal dynamics, topology extraction

## Abstract

Granger causality (GC) is a powerful method for causal inference for time series. In general, the GC value is computed using discrete time series sampled from continuous-time processes with a certain sampling interval length τ, i.e., the GC value is a function of τ. Using the GC analysis for the topology extraction of the simplest integrate-and-fire neuronal network of two neurons, we discuss behaviors of the GC value as a function of τ, which exhibits (i) oscillations, often vanishing at certain finite sampling interval lengths, (ii) the GC vanishes linearly as one uses finer and finer sampling. We show that these sampling effects can occur in both linear and non-linear dynamics: the GC value may vanish in the presence of true causal influence or become non-zero in the absence of causal influence. Without properly taking this issue into account, GC analysis may produce unreliable conclusions about causal influence when applied to empirical data. These sampling artifacts on the GC value greatly complicate the reliability of causal inference using the GC analysis, in general, and the validity of topology reconstruction for networks, in particular. We use idealized linear models to illustrate possible mechanisms underlying these phenomena and to gain insight into the general spectral structures that give rise to these sampling effects. Finally, we present an approach to circumvent these sampling artifacts to obtain reliable GC values.

## 1. Introduction

There have been great advances in experimental observational techniques in neuroscience, from single-unit, multi-unit, local field potential (LFP), to non-invasive electroencephalography (EEG), magnetoencephalography (MEG), and functional Magnetic Resonance Imaging (fMRI). These experimental tools have expanded the examination of physiological correlations of neuronal responses to stimuli to causal effects of how different parts of the nervous system influence one another. One of the fundamental questions is how one can infer causal relations between neurons or between neuronal populations through measured time series, which represent neural activities of different neurons or neuronal populations.

There is a long history of studying causal relations between time series. Granger causality (GC) has been developed to study how one time series is influencing the other. Recently GC has been applied to extract causal or structural information from time series (Strogatz, 2001; Boccaletti et al., 2006; Bressler and Seth, 2011). It has been applied to analyze times series obtained through invasive techniques, such as single-unit, multi-unit (Passaro et al., 2008), LFP (Brovelli et al., 2004; Bressler et al., 2007) recordings as well as non-invasive techniques, such as EEG (Astolfi et al., 2006), MEG (Gow et al., 2008), and fMRI (Roebroeck et al., 2005; Deshpande et al., 2008; Hamilton et al., 2010). The GC theory aims to analyze causal influence of one time series *X*_*t*_ on the other *Y*_*t*_ by examining whether the prediction of *Y*_*t*_ can be improved upon the incorporation of information of *X*_*t*_ (Wiener, 1956; Granger, 1969; Geweke, 1982). Although GC has proven to be a powerful framework to study directed causal connectivity within the brain, there are many challenges in its application to acquire reliable results. Usually the application of GC requires the time series to be linear. When time series are linear and Gaussian, GC is equivalent to the transfer entropy (Barnett et al., 2009; Bressler and Seth, 2011). For non-linear, non-Gaussian time series, it remains an active research field to study the applicability of the GC. Note that, the notion of GC is statistical rather than structural, i.e., through statistical features of responses, it identifies directed statistical functional connectivity, which may be different from physical causal interactions in the systems.

As a statistical method, one of the important issues in the GC analysis is how to sample the dynamics in order to obtain suitable time series for a reliable result from GC analysis. It has been pointed out that GC is not invariant to the sampling interval (McCrorie and Chambers, 2006). Spurious causality may arise if sampling is not sufficiently fine to capture the evolution of dynamical variables (McCrorie and Chambers, 2006). In its application to the analysis of fMRI data, one is confronted with the unavoidable issue of low-pass filtering and down-sampling in processing the BOLD signal (Roebroeck et al., 2005; Danks and Plis, 2013).

However, the directed statistical functional connectivity as revealed in the GC analysis is intimately related to the structural connectivity of networks. Previously, we have applied the GC analysis to reconstruct network structural connectivity and our results demonstrate that the GC analysis can be successfully used to reconstruct the coupling structures of the integrate-and-fire (I&F) networks (Zhou et al., 2013b, 2014). We note in passing that for smoothly coupled oscillator networks (with or without noisy inputs), techniques including chaotic synchronization or phase dynamics have been used to uncover the structural connectivity via random phase reset or their responses to perturbations (Yu et al., 2006; Timme, 2007; Napoletani and Sauer, 2008; Smirnov and Bezruchko, 2009; Ren et al., 2010; Levnajić and Pikovsky, 2011), whereas, for some simple non-smooth network dynamics, e.g., δ-pulse coupled, current-based integrate-and-fire systems, their topologies can be reconstructed by designing a specific set of external inputs (Bussel et al., 2011). However, in general, it remains a challenging task to directly uncover the network topology for a non-smooth, non-linear system, such as the conductance-based I&F networks, given the condition that only the dynamical activity at individual nodes can be measured. The GC analysis in our network construction uses time series measured from the network dynamics response, such as the membrane potential, to a natural input. In this approach, we do not design specific inputs to probe the specific response of the system in order to uncover the network topology.

Because most dynamical quantities are continuous in time, GC values are computed using the discrete time series sampled from these continuous time processes. In this work, we analyze sampling artifacts in the GC analysis. In particular, we examine the reliability of the I&F network topology reconstructed using the GC analysis, by studying behaviors of the GC value as a function of sampling interval length. We will term this function as the GC sampling structure. As will be shown below, surprisingly, there are oscillations in the GC sampling structure and the GC may even vanish on a set of finite sampling interval lengths even if there are physical couplings between neurons. The phenomenon of vanishing GC on a set of sampling interval lengths obviously complicates the interpretation of causal inference and raises the issue of the reliability of network topology reconstruction using the GC analysis. In addition, if sampling interval is not sufficiently small, as mentioned above, GC may not vanish even when there is actually no causal influence (McCrorie and Chambers, 2006). Therefore, in general, without properly taking into account the sampling effect, different conclusions can be drawn about causality, due to the difference in sampling, when the GC analysis is applied to experimental observational data. One may presume that if we used discrete time series sampled using ever finer interval lengths from a continuous process in time, a more reliable GC value for causal inference could be obtained since the sampled time series in such case is more and more close to the continuous dynamical process. However, as we will show below, the GC value actually approaches zero linearly in proportion to the sampling interval length. Therefore, in the GC analysis for the network topology reconstruction, one of the main theoretical challenges is what is an appropriate sampling interval in order to obtain reliable GC values.

To answer the question of how to determine the sampling interval, we first characterize various features associated with the GC sampling structures arising from the network topology reconstruction. We will use idealized linear models to illustrate possible mechanisms underlying these structures and to gain insight into reliability of the GC analysis applied to linear and non-linear systems. Finally, we will present general strategies to circumvent these complications in the GC analysis. It is important to point out that these sampling issues arise from how discrete time series for the GC analysis are sampled from continuous time processes and they need to be addressed regardless of whether one uses a parametric method or a non-parametric method for the GC evaluation (Geweke, 1982; Dhamala et al., 2008).

This article is organized as follows. In the section of Methods, we briefly introduce the I&F network model, the GC analysis and its application in the network reconstruction. In the section of Results, first, we numerically study the GC sampling structures for the I&F networks. We observe that GC values oscillate with respect to the sampling interval length τ and approach zero linearly in proportion to τ as τ tends to zero. These two phenomena may give rise to complications for reliable GC analysis of the I&F network, in addition to the possible spurious GC in the absence of causal influence. Second, we study mechanisms of oscillations in GC sampling structures through linear models. Third, we study the limiting behavior of GC as τ tends to zero and discuss the underlying mechanisms. In the section of Discussion and Conclusion, we describe our approach of eliminating the sampling artifacts in the application of GC and present our conclusions. In Appendices A–C of Supplementary Material, we present related mathematical details.

## 2. Materials and methods

### 2.1. I&F network model

As we mentioned in the Introduction, we have applied GC analysis to reconstruct the topological connectivity of I&F networks. Experimentally, it has been shown that models of I&F neurons are able to capture rather well the subthreshold dynamics of a real neuron in terms of linear response properties as well as statistical firing properties of a real neuron (Carandini et al., 1996; Rauch et al., 2003; Burkitt, 2006). The models of I&F neurons have been extensively used in simulations of large-scale neuronal networks in the modeling of cortical phenomena in the brain (Somers et al., 1995; Troyer et al., 1998; McLaughlin et al., 2000; Tao et al., 2004; Cai et al., 2005; Rangan et al., 2005; Zhou et al., 2013a). In this work, we study the network of the conductance-based, I&F neurons of excitatory type and its dynamics is governed by the following equations:

(1) $$\{\begin{array}{l} \frac{\text{d}V_{i}}{\text{d}t}=-G_{\text{L}}\text{​}\left(V_{i}-\epsilon_{\text{L}}\right)-G_{i}\text{​}\left(V_{i}-\epsilon_{\text{E}}\right),\\ \frac{\text{d}G_{i}}{\text{d}t}=-\frac{1}{\sigma }G_{i}+\sum_{j\;\neq \;i}^{N}\sum_{k}s_{ji}\delta \text{​}\left(t-T_{j,k}\right)+\lambda \sum_{l}\delta \text{​}\left(t-T_{i,l}^{\text{P}}\right), \end{array}$$

where *V*_*i*_ and *G*_*i*_ are the membrane potential and conductance for the *i*th neuron in the network, respectively, *G*_*L*_ is the leak conductance, ϵ_L_ is the resting potential, ϵ_E_ is the reversal potential, σ is the conductance time constant. When *V*_*i*_ is between the threshold *V*_th_ and the resting potential ϵ_L_, the dynamics of a neuron is described by Equation (1). However, when the membrane potential of a neuron reaches the threshold *V*_th_, it is reset to the resting potential ϵ_L_ for a certain period τ_ref_ (refractory period). An action potential of a real neuron is modeled here by the event of the threshold crossing and the voltage resetting. The event of this threshold-reset dynamics is referred to as a firing event (spike) of a neuron. *s*_*ji*_ is the connection strength from the presynaptic neuron *j* to the postsynaptic neuron *i*. For simplicity, we study a homogeneously coupled network, i.e., *s*_*ji*_ = *sA*_*ji*_, where *s* is the connection strength, **A** = (*A*_*ji*_) is the adjacency matrix of the network. *T*_*j*,*k*_ is the time of the *k*th spike of the *j*th neuron, whereas *T*^*P*^_*i,l*_ is the arrival time of the *l*th spike of the input to the *i*th neuron. The input is a Poisson process with rate ν and strength λ.

In our work, we use dimensionless unit for membrane potentials, in particular, *V*_th_ = 1, ϵ_L_ = 0, ϵ_E_ = $\frac{14}{3}$. These correspond to unscaled physiological values, *V*_th_ = −55 mV, ϵ_L_ = −70 mV, and ϵ_E_ = 0 mV (McLaughlin et al., 2000; Cai et al., 2005; Rangan et al., 2005; Zhou et al., 2013a). Time constants retain its dimension, for which we use ms for its unit. We set τ_ref_ = 2 ms and the conductance time constant σ = 2 ms. Conductance has the unit ms^−1^. We have *G*_L_ = 0.05 ms^−1^, which corresponds to the physiological membrane time constant 20 ms. The method we used to solve system (1) numerically is a fourth order Runge-Kutta method with spike-spike corrections, the details of which can be found in Rangan and Cai (2007).

In addition to voltage signals, we also use time series constructed from spike trains:

$$S(t)=\sum_{i\;=\;1}^{+\infty }\delta \text{​}\left(t-T_{\text{f}}^{i}\right),$$

where, δ(*t* − *T*^*i*^_f_) is the Dirac delta function, *T*^*i*^_f_ is the firing time of the *i*th spike. In order to avoid the infinity in the Dirac δ-function, we convolve these spikes with a kernel. The simplest kernel is the indicator function with width *d*. Then, a spike train can be represented as the following:

(2) $$\tilde{S(t)}=\int_{0}^{+\infty }\sum_{i\;=\;1}^{+\infty }\delta \text{​}\left(\tau -T_{\text{f}}^{i}\right)I_{d}\text{​}\left(t-\tau \right)\text{​d}\tau ,$$

where, *I*_*d*_(*x*) = 1 for |*x*| ≤ $\frac{d}{2}$ and *I*_*d*_(*x*) = 0 for |*x*| > $\frac{d}{2}$. We use sufficiently small *d* so there are no overlaps between spikes. Then, we can obtain a time series of binary type by sampling signal (2) with a certain sampling interval length.

In the following, we will perform the GC analysis on the time series of voltage or spike trains to reconstruct the I&F network topology, i.e., the adjacency matrix **A** = (*A*_*ji*_) and discuss the sampling issues in this approach.

### 2.2. Brief introduction to the GC analysis

The GC analysis is based on the idea that, if one can reduce the prediction error about a time series after incorporating the history of the second time series, then there must be a causal influence of the second time series on the first (Wiener, 1956; Granger, 1969; Geweke, 1982). The GC analysis can be naturally formulated in a bivariate form. However, the causality analysis can also be naturally generalized to a multivariate form using conditional Granger causality (Geweke, 1984; Ding et al., 2006). In the following, for ease of later discussions, we briefly recapitulate properties of Granger causality in the bivariate setting.

For two stationary time series *X*_*t*_ and *Y*_*t*_, one can perform autoregression to obtain

(3) $$\{\begin{array}{ll} X_{t} & =\sum_{j\;=\;1}^{\infty }a_{1j}X_{t\;-\;j}+\epsilon_{1t},\\ Y_{t} & =\sum_{j\;=\;1}^{\infty }d_{1j}Y_{t\;-\;j}+\eta_{1t}, \end{array}$$

where ϵ_1*t*_ and η_1*t*_ are autoregression residuals representing the prediction errors when we consider the history of each time series separately. Here, Σ_1_ = var(ϵ_1*t*_), Γ_1_ = var(η_1*t*_). The corresponding joint regression has the following form for times series *X*_*t*_ and *Y*_*t*_

(4) $$\{\begin{array}{ll} X_{t} & =\sum_{j\;=\;1}^{\infty }a_{2j}X_{t\;-\;j}+\sum_{j\;=\;1}^{\infty }b_{2j}Y_{t\;-\;j}+\epsilon_{2t},\\ Y_{t} & =\sum_{j\;=\;1}^{\infty }c_{2j}X_{t\;-\;j}+\sum_{j\;=\;1}^{\infty }d_{2j}Y_{t\;-\;j}+\eta_{2t}, \end{array}$$

where ϵ_2*t*_ and η_2*t*_ are joint regression residuals representing the prediction errors after we consider a shared history for both time series. The covariance matrix for ϵ_2*t*_ and η_2*t*_ is denoted by

$$\Sigma =\left[\begin{array}{cc} \Sigma_{2} & {ϒ}_{2}\\ {ϒ}_{2} & \Gamma_{2} \end{array}\right],$$

where Σ_2_ = var(ϵ_2*t*_), Γ_2_ = var(η_2*t*_), ϒ_2_ = cov(ϵ_2*t*_, η_2*t*_). By the Gauss-Markov theorem (Scheffé, 1959), the series {ϵ_1*t*_}, {ϵ_2*t*_}, {η_1*t*_}, and {η_2*t*_} are white noise time series. It is evident from Equations (3) and (4) that Σ_2_ ⩽ Σ_1_ and Γ_2_ ⩽ Γ_1_, that is, one can never obtain a worse prediction (i.e., greater residual variance) of one time series after incorporating information from the other time series.

Following the idea of Granger causality, if Σ_2_ = Σ_1_, i.e., the prediction error cannot be reduced by the joint regression, then there is no causal influence from *Y*_*t*_ to *X*_*t*_. However, if Σ_2_ < Σ_1_, there is a causal influence from *Y*_*t*_ to *X*_*t*_. This causal influence is characterized by the Granger causality which is defined as

(5) $$F_{y\to x}=\ln\frac{\Sigma_{1}}{\Sigma_{2}}.$$

Clearly, *F*_*y*→*x*_ ⩾ 0 because Σ_2_ ⩽ Σ_1_; *F*_*y*→*x*_ = 0 when Σ_2_ = Σ_1_. Similarly, Granger causality from {*X*_*t*_} to {*Y*_*t*_} is defined as

(6) $$F_{x\to y}=\ln\frac{\Gamma_{1}}{\Gamma_{2}}.$$

The idea of instantaneous causality is to quantify the mutual instantaneous influence of both time series without any time lag. If ϒ_2_ = 0, i.e., ϵ_2*t*_ and η_2*t*_ are uncorrelated, then incorporation of the new instantaneous information of *X*_*t*_ (ϵ_2*t*_) cannot help to reduce the variance of η_2*t*_ and vice versa. Therefore, the instantaneous causality can be defined as

(7) $$F_{x\;\cdot \;y}=\ln\frac{\Gamma_{2}\Sigma_{2}}{\left|\Sigma \right|},$$

where |Σ| is the determinant of the matrix Σ. Note that, when ϒ_2_ = 0, |Σ| = Γ_2_Σ_2_ − ϒ^2^_2_ = Γ_2_Σ_2_, therefore *F*_*x*.*y*_ vanishes, i.e., no instantaneous causality. The total Granger causality between {*X*_*t*_} and {*Y*_*t*_} can now be defined as

$$F_{x\;,\;y}=\ln\frac{\Sigma_{1}\Gamma_{1}}{\left|\Sigma \right|},$$

which can easily be seen to be the sum of all the three causality terms Equations (5), (6), and (7):

(8) $$F_{x,y}=F_{y\to x}+F_{x\to y}+F_{x\;\cdot \;y}.$$

Note that, in the GC analysis of our network topology reconstruction, we mainly focus on the values of *F*_*y*→*x*_ and *F*_*x*→*y*_ because they quantify the directional causal strengths. As can be seen below, *F*_*y*→*x*_ and *F*_*x*→*y*_ can be related to the directional connectivity of a network (Zhou et al., 2013b, 2014).

For our later discussions, in addition to the time-domain description of GC, we also briefly summarize the description of GC in the frequency domain (Geweke, 1982; Ding et al., 2006). For the bivariate time series *X*_*t*_, *Y*_*t*_, the spectral matrix **S**(ω) is represented as $S(\omega )=\left[\begin{array}{cc} S_{xx}(\omega ) & S_{xy}(\omega )\\ S_{yx}(\omega ) & S_{yy}(\omega ) \end{array}\right]$, where *S*_*xx*_(ω) is the auto-spectrum of the time series *X*_*t*_, defined by $S_{xx}(\omega )=\sum_{n\;=\;-\infty }^{+\infty }\text{cov}(X_{t},X_{t\;-\;n})e^{-in\omega }$, *S*_*yy*_(ω) is the auto-spectrum of the time series *Y*_*t*_, *S*_*xy*_(ω) is the cross-spectrum of *X*_*t*_ and *Y*_*t*_, defined by $S_{xy}(\omega )=\sum_{n\;=\;-\infty }^{+\infty }\text{cov}(X_{t},Y_{t\;-\;n})e^{-in\omega }$. Note that *S*_*yx*_(ω) is the complex conjugate of *S*_*xy*_(ω). The covariances of residuals possess the following spectral representations [see (Geweke, 1982; Ding et al., 2006) for details]:

(9) $$\ln\Sigma_{1}=\frac{1}{2\pi }\int_{-\pi }^{\pi }\ln S_{xx}(\omega )\text{d}\omega ,$$

(10) $$\ln\Gamma_{1}=\frac{1}{2\pi }\int_{-\pi }^{\pi }\ln S_{yy}(\omega )\text{d}\omega ,$$

(11) $$\ln\left|\Sigma \right|=\frac{1}{2\pi }\int_{-\pi }^{\pi }\ln\left|S(\omega )\right|\text{d}\omega ,$$

(12) $$F_{x,y}=\frac{1}{2\pi }\int_{-\pi }^{\pi }\ln\left(\frac{S_{yy}(\omega )S_{xx}(\omega )}{\left|S(\omega )\right|}\right)\text{d}\omega ,$$

where |**S**(ω)| = *S*_*xx*_(ω)*S*_*yy*_(ω) − *S*_*xy*_(ω)*S*_*yx*_(ω) is the determinant of the spectral matrix **S**(ω).

### 2.3. Extraction of the network topology

**Figure 1:**
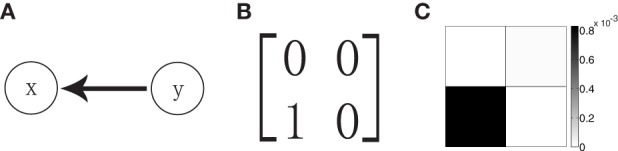
**(A)** Schematic representation of a unidirectional two-neuron network with only neuron *y* synaptically connected to the postsynaptic neuron *x*. **(B)** The corresponding adjacency matrix **A** with *A*_*yx*_ = 1 for the network in **(A)**. **(C)** The grayscale grid representation of the GC matrix **F** (*F*_*ij*_ = *F*_*i* → *j*_) for the network. The gray scale bar indicates the value of GC. The parameters of the network are ν = 1ms^−1^, λ = 0.012, and *s* = 0.02.

We now turn to the reconstruction of the network topology using the GC analysis. For a two-neuron network as shown in Figure 1A in which neuron *x* is postsynaptic to neuron *y* whereas there is no synaptic input from neuron *x* to neuron *y*, the corresponding adjacency matrix of the network is shown in Figure 1B. Applying the GC analysis on voltage time series with sampling interval length τ = 0.5 ms, time length *T* = 10^6^ ms and regression order *p* = 20, we obtain *F*_*y*→*x*_ = 8.3 × 10^−4^ and *F*_*x*→*y*_ = 0.1 × 10^−4^ (Figure 1C). Note that, in our work, the Bayesian information criterion (Schwarz, 1978) is used to determine the regression order *p*. In our example in Figure 1, we have *F*_*y*→*x*_ » *F*_*x*→*y*_ and the GC value from neuron *y* to neuron *x* is almost two orders of magnitude larger than the inverse direction. From this, one may conclude that GC values are linked to the adjacency matrix of the network, i.e., *A*_*yx*_ = 1 and *A*_*xy*_ = 0. This is also consistent with the result by performing statistical significance test (see Appendix C in Supplementary Material for details) (Zhou et al., 2013b, 2014). In the present work, we focus on the implication of sampling effects and on the assessment of the reliability of this reconstruction by the GC analysis.

## 3. Results

### 3.1. Effects of sampling

As discussed in the Introduction, the GC analysis has emerged as a popular tool in detecting causal relations among times series of physiological data measured in the brain. Note that most of the dynamical quantities underlying these times series are continuous in time, therefore, a natural and important issue arises about what is the proper way of constructing discrete time series from continuous quantities when applying the GC analysis. To address this issue, we examine the following two related questions: one is whether it makes great differences when discrete time series sampled at different time intervals are used and the other is whether the GC analysis would become more reliable as the sampling interval length shrinks to zero. We will answer these two questions using our I&F network dynamics below. Due to its own importance, as mentioned previously, we will refer to the GC value as a function of sampling interval length τ as *the GC sampling structure*.

#### 3.1.1. Oscillations in the GC sampling structure

In the topology extraction for the network dynamics of two neurons (see Methods) with unidirectional interactions, whose topology is shown in Figure 1A, we perform the GC analysis on both voltage and spike train signals of the neurons. In order to examine possible differences of GC values with different sampling intervals, we first perform a scan of interval lengths in sampling of the dynamical variables from the I&F network. Note that we evaluate the GC values with different sampling interval lengths based on the same original signal, i.e., the same total duration of the signal.

**Figure 2:**
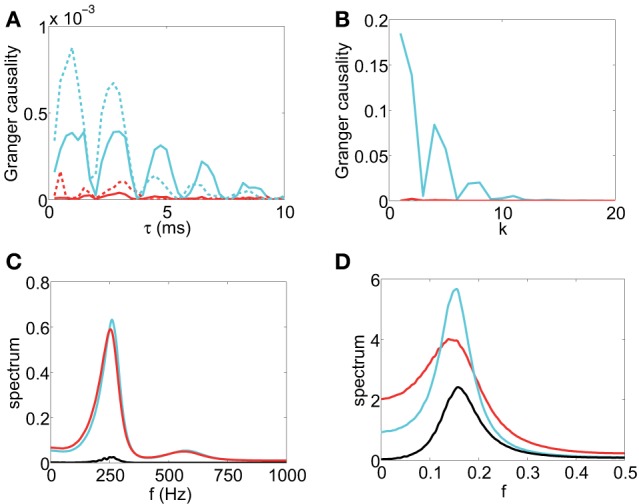
**The GC sampling structure and corresponding spectra**. The top row of panel is GC vs. sampling interval length: **(A)** *F*_*x* → *y*_ (red), *F*_*y* → *x*_ (cyan) obtained from voltage time series and *F*_*x* → *y*_ (red dash), *F*_*y* → *x*_ (cyan dash) obtained from spike train time series with sampling interval τ. The time series are generated by the I&F network whose topology is shown in Figure 1A with parameters ν = 1ms^−1^, λ = 0.066, *s* = 0.02. **(B)** *F*_*x* → *y*_ (red) and *F*_*y* → *x*_ (cyan) with sampling interval length *k* (see text) for the second order autoregressive model (13). **(C)** and **(D)**: Corresponding spectra of the voltage time series for **(A)** and the autoregressive time series for **(B)**, respectively: *S*_*xx*_ (cyan), *S*_*yy*_ (red), |*S*_*xy*_| (black).

The scanning result is summarized in Figure 2A, which displays the GC value as a function of sampling interval length τ for the I&F dynamics. From Figure 2A, for GC values obtained from both the voltage and spike train time series, we have the following Observations: (i) there are oscillations in the GC sampling structure, (ii) the GC value becomes almost zero periodically, and (iii) the GC value oscillates almost at a constant frequency (approximately 500 Hz in Figure 2A), (iv) the GC value decays to zero as τ becomes sufficiently large. It is easy to appreciate Observation (iv) because for time series with a sufficiently large sampling interval length, much of the information is lost, resulting in unreliable causal detection, i.e., vanishing GC values. These observations demonstrate that for I&F dynamics there are great effects of sampling interval length on the GC values regardless of whether the voltage or spike train time series are used.

In general, there are oscillations, with a narrow spectral band or a broad spectral band, in time series from the I&F networks. It is natural to use the frequency domain to examine these features of the time series. We ask whether there is a relation between the oscillations of time series which can be revealed by the spectra and the GC sampling structure. In Figure 2C, the peak frequencies of all spectra *S*_*xx*_, |*S*_*xy*_| and *S*_*yy*_ are approximately 250 Hz which is half of the oscillation frequency 500 Hz in the GC sampling structure in Figure 2A. In the following, we will investigate this relation.

From the results of Figure 2A, a question naturally arises: whether the oscillation phenomenon is general and is not confined to the time series from the non-linear dynamics of the I&F networks. In particular, we ask whether the oscillations exist for *linear* autoregressive models, for which the GC framework is established. In order to answer this question, we study a simple example of linear models. A representative second-order autoregressive model we study is

(13) $$\{\begin{array}{l} X_{t}=0.9X_{t\;-\;1}-0.6X_{t\;-\;2}-0.3Y_{t\;-\;1}+0.15Y_{t\;-\;2}+\epsilon_{t},\\ Y_{t}=0.7Y_{t\;-\;1}-0.4Y_{t\;-\;2}+\eta_{t}, \end{array}$$

where ϵ_*t*_ and η_*t*_ are Gaussian white noise time series with variances var(ϵ_*t*_) = 0.5, var(η_*t*_) = 1 and covariance cov(ϵ_*t*_, η_*t*_) = 0.2. Figure 2B displays the GC values computed using the time series generated by the model (13) with different “sampling interval length” τ = *k*, i.e., we use every datum point after skipping *k* − 1 points in between. From Figure 2B, we can also observe the oscillatory feature in the GC sampling structure, which also exhibits the behavior of GC that almost periodically vanishes. Note that we have examined many other parameters for second-order autoregressive models and found that the oscillatory features are quite common, as will be addressed below. In Figure 2D, the peak frequencies of all spectra for this model are approximately $\frac{1}{6}$ which is again half of the oscillation frequency $\frac{1}{3}$ in the GC sampling structure in Figure 2B.

In summary, it can be seen that the oscillations of the GC values with respect to sampling interval length is a common feature, not only for I&F networks but also for linear models. These features in the GC sampling structure have strong ramifications in the application of the GC analysis. Importantly, it is disconcerting to observe that the GC value almost become zero at some sampling interval lengths despite the fact that there are clearly physical connections in the network or the coupling in the linear model between the two time series. We further note that, in Figure 2A, *F*_*x*→*y*_ from both voltage and spike train time series displays non-zero values for some ranges of τ, in contrast to the physical couplings in the system for which there is no synaptic connection from neuron *x* to *y*. As will be shown below, this spurious GC also arises for linear systems. Evidently, the phenomena of vanishing GC and spurious GC will greatly complicate the reliability of any conclusions about the causal influence and will make the validity of the network topology reconstruction questionable. Later, we will return to the resolution of these issues.

#### 3.1.2. The GC sampling structure as τ→0

**Figure 3:**
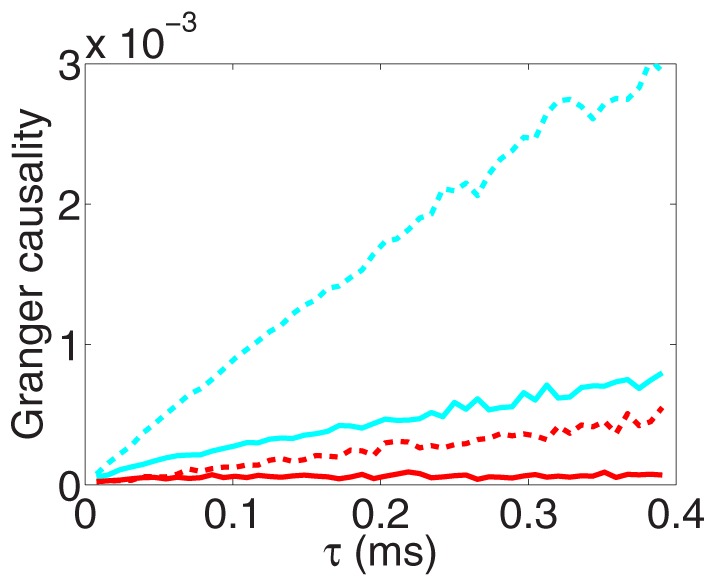
**The GC sampling structure as sampling interval length tends to zero**. *F*_*x*→*y*_ (red), *F*_*y*→*x*_ (cyan) obtained from voltage time series and *F*_*x*→*y*_ (red dash), *F*_*y*→*x*_ (cyan dash) obtained from spike train time series with sampling interval length τ. Note that, by the asymptotic distribution theory of GC (Geweke, 1982), the estimator of a directed GC has a bias $\frac{p}{n}$, where *p* is the regression order and *n* is the length of the discrete time series. We have used the Bayesian information criterion (Schwarz, 1978) to determine the regression order *p* and have subtracted this type of biases in the figure (see Appendix C in Supplementary Material for details). The time series are generated by the I&F network whose topology is shown in Figure 1A with parameters ν = 1 ms^−1^, λ = 0.0177, *s* = 0.02.

As demonstrated through the oscillation phenomenon of GC above, clearly, we cannot choose sampling interval length arbitrarily in the application of GC analysis. Then, an important issue arises: what should be the criteria of choosing a correct sampling interval length to obtain discrete time series in measurement for reliable GC analysis. For a natural discrete-time dynamics, we can simply use their intrinsic intervals, e.g., for discrete time series generated by an autoregressive model, and we can then obtain reliable GC values for causal interpretations because the entire information is incorporated in the causal analysis. However, most physical quantities are continuous in time, one does not have particular intrinsic intervals for sampling. In order to obtain reliable GC values, one possible scenario, similar to the discrete case, is that it is always better if one uses more finely sampled time series because apparently more information is incorporated for causality determination. To examine this scenario, we study the convergence property of the GC sampling structure as the sampling interval length τ tends to 0. The corresponding numerical results are shown in Figure 3 for a two-neuron I&F network.

From Figure 3, we observe that for both voltage and spike train time series, GC values approach 0 almost *linearly* as the sampling interval length τ tends to 0. This phenomenon of vanishing GC values implies that we eventually fail to extract real causal relation through GC analysis when sampling interval length vanishes regardless of whether or not there are interactions between two time series. In this situation, obviously, we cannot use the limit GC value as τ → 0 to infer the causal interaction especially for the I&F networks. Here, we note that this limit effect of sampling is general for continuous-time processes and the underlying mechanism will be demonstrated later.

In summary, the phenomena shown in Figures 2, 3 demonstrate that time series obtained by using different sampling interval lengths lead to great differences in GC values and a finer sampling does not result in more reliable GC values for causal interpretation. Therefore, in order to obtain meaningful interpretation through GC, how to choose a sampling interval length becomes an important issue in practice. Without properly taking this issue into account, GC analysis may produce unreliable or even opposite conclusions about causal influence when applied to empirical data sampled with different sampling interval lengths. In what follows, we first use linear regressive models and spectral methods to study theoretically possible mechanisms underlying the oscillation phenomenon. With these idealized models we can gain insight into possible origins of general oscillations in the GC sampling structure.

### 3.2. Mechanism of oscillations in the GC sampling structure

#### 3.2.1. Spectra and the GC sampling structure

We will invoke the frequency domain description of GC for our analysis of the GC sampling structure. First, we discuss the relation between the spectrum *S*(ω) for the time series {*X*_0_, *X*_1_, *X*_2_, …} whose sampling time interval is τ, and spectrum *S*^(*k*)^(ω) for time series {*X*_0_, *X*_*k*_, *X*_2*k*_, …}, which is a time series sampled at the interval length *k*τ, *k* being a positive integer. Using the Wiener-Khinchin theorem, we can relate the spectra *S*(ω) and *S*^(*k*)^(ω) of these two time series to their time correlations, thus enabling us to show that

(14) $$S^{\left(k\right)}(\omega )=\frac{1}{k}\sum_{j\;=\;0}^{k\;-\;1}S(\frac{\omega }{k}+\frac{2\pi j}{k}).$$

The details of the proof can be found in Appendix A in Supplementary Material. Note that Equation (14) is also valid for cross-spectrum *S*_*xy*_(ω) of time series *X*_*t*_ and *Y*_*t*_, which is defined by $S_{xy}(\omega )=\sum_{n\;=\;-\infty }^{+\infty }\text{cov}(X_{t},Y_{t\;-\;n})e^{-\;in\omega }$.

A direct implication of Equation (14) is that if *S*(ω) = *C* (*C* is a constant), then *S*^(*k*)^(ω) ≡ *C*. That is, if the original time series is white, then the time series obtained by sampling with any interval length remains to be white. In addition, we have the following results about GC in the case in which one of the bivariate time series is white and the other has no correlation with it. If *Y*_*t*_, *t* is an integer, is a white noise series and cov(*Y*_*t*_, *X*_*t* − *i*_) = 0 for any positive integer *i* > 0, then *F*^(*k*)^_*x*→*y*_ ≡ 0. In particular, when cov(*Y*_*t*_, *X*_*t* − *i*_) = 0 for any integer *i* ⩾ 0, *F*^(*k*)^_*x*→*y*_ ≡ *F*^(*k*)^_*x*·*y*_ ≡ 0. These facts conform with our intuition that there is no causal flow if there is no correlation between *X*_*t*_ and *Y*_*t*_ and the *Y*_*t*_ remains always white (see Appendix A in Supplementary Material for details). We will use these facts in our discussions below.

We now turn to the question of what is the structure in time series that will give rise to oscillations in the GC sampling structure. A possible origin of oscillations in the GC sampling structure may be a consequence of oscillations in the original time series. Our strategy of tackling this question is as follows: First, we note that the spectrum of the original time series characterizes the oscillation strength of the time series over different frequencies. We will construct simplified models in which we can focus on the relationship between the peak frequencies of the spectrum and oscillations in the GC sampling structure. We will construct different classes of models which allow us to directly analyze the GC sampling structure through the spectral representation (14). Using Equation (14), we can derive an expression of the spectral matrix **S**^(*k*)^ with sampling interval *k*, $S^{\left(k\right)}=\left[\begin{array}{cc} S_{xx}^{\left(k\right)} & S_{xy}^{\left(k\right)}\\ S_{yx}^{\left(k\right)} & S_{yy}^{\left(k\right)} \end{array}\right]$, and **S**^(*k*)^ can be directly used to compute the total causality *F*^(*k*)^_*x*,*y*_ through Equation (12) as follows

(15) $$F_{x,y}^{\left(k\right)}=-\frac{1}{2\pi }\int_{-\pi }^{\pi }\ln\left(1-\frac{S_{xy}^{\left(k\right)}(\omega )S_{yx}^{\left(k\right)}(\omega )}{S_{xx}^{\left(k\right)}(\omega )S_{yy}^{\left(k\right)}(\omega )}\right)\text{d}\omega .$$

Because the total causality in Equation (8) involves three different terms and it is usually difficult to derive the analytical formula for each term, one way of tackling this issue is to consider each term separately. In particular, we construct a special model for which we have *F*^(*k*)^_*x*→*y*_ ≡ *F*^(*k*)^_*x.y*_ ≡ 0. Then, the directional GC *F*^(*k*)^_*y*→*x*_ is identical to the total causality *F*^(*k*)^_*x*,*y*_ and we can therefore analyze the GC sampling structure *F*^(*k*)^_*y*→*x*_ through the total causality in Equation (15). If the condition *S*^(*k*)^_*xx*_(ω)*S*^(*k*)^_*yy*_(ω) » *S*^(*k*)^_*xy*_(ω)*S*^(*k*)^_*yx*_(ω) holds, we can obtain the following simplified approximation for the directional GC *F*^(*k*)^_*y*→*x*_:

(16) $$F_{y\to x}^{\left(k\right)}=\frac{1}{2\pi }\int_{-\pi }^{\pi }\frac{S_{xy}^{\left(k\right)}(\omega )S_{yx}^{\left(k\right)}(\omega )}{S_{xx}^{\left(k\right)}(\omega )S_{yy}^{\left(k\right)}(\omega )}\text{d}\omega .$$

In the following, we will use simplified models to reveal the origin of oscillations in the GC sampling structure, thus, providing insight into an intuitive understanding of the possible origin of oscillations in the GC sampling structure for the I&F network as well as for other general situations.

#### 3.2.2. Idealized model I

The first idealized linear model we have constructed is as follows

(17) $$\{\begin{array}{ll} X_{t} & =a(L)\epsilon_{t}+b(L)\eta_{t},\\ Y_{t} & =\eta_{t}, \end{array}$$

where {ϵ_*t*_} and {η_*t*_} are independent white noise series with covariance matrix $\Sigma =\left[\begin{array}{cc} \mu & 0\\ 0 & 1 \end{array}\right]$. *L* is the lag operator satisfying *LX*_*t*_ = *X*_*t* − 1_. *a*(*L*) = 1 + *a*_1_*L* + *a*_2_*L*^2^ + …, *b*(*L*) = *b*_1_*L* + *b*_2_*L*^2^ + …. We can obtain the spectra of *X*_*t*_, *Y*_*t*_, which are

$$\begin{array}{l} S_{xx}=a(\omega )a^{*}(\omega )\mu +b(\omega )b^{*}(\omega ),\\ S_{yy}=1, \end{array}$$

i.e., *Y*_*t*_ is a white noise time series, and

$$S_{xy}=b(\omega ),$$

where $a(\omega )=1+\sum_{n\;=\;1}^{+\infty }a_{n}e^{-\;in\omega }$ and $b(\omega )=\sum_{n\;=\;1}^{+\infty }b_{n}e^{-in\omega }$, which are the Fourier transforms of *a*(*L*) and *b*(*L*), respectively.

By the result of the previous section (also see Appendix A in Supplementary Material for details), we have *F*^(*k*)^_*x*·*y*_ ≡ *F*^(*k*)^_*x*→*y*_ ≡ 0 for this model, therefore, the other directional GC is equal to the total causality, i.e., *F*^(*k*)^_*y*→*x*_ ≡ *F*^(*k*)^_*x*,*y*_. Then, we can use Equations (15) and (16) to analyze the relation between *F*^(*k*)^_*y*→*x*_ and sampling interval length *k*.

To illustrate clearly the sampling phenomena, we discuss two classes of *a*(*L*) and *b*(*L*) in the following.

For the first class, we fix *S*_*xx*_ = *a*(ω)a^*^(ω)μ + *b*(ω)b^*^(ω) = *C*, where *C* is constant and we always assume *C* » max(*b*(ω)b^*^(ω)) so as to guarantee that Equation (16) is a good approximation. Under this condition, there are no oscillations in the time series *X*_*t*_ as discussed previously and the only spectrum that varies over *k* is the cross-spectrum *S*_*xy*_. Then, we can find explicit asymptotic expressions and approximations of *F*^(*k*)^_*y*→*x*_ in order to study the relation between the peak frequency of *S*_*xy*_(ω) and the oscillations of *F*^(*k*)^_*y*→*x*_ with respect to the sampling interval length *k*. The spectrum *S*_*xy*_ corresponds to the Fourier transform of the correlation function between *X*_*t*_ and *Y*_*t*_. In applications, it is common that a correlation function has damping and oscillation structures. Therefore, we will study the following three cases in the first class:

Case 1.1 $b(L)=\sum_{j\;=\;1}^{+\infty }e^{-\tau_{d}j}L^{j}$.
Case 1.2 $b(L)=\sum_{j\;=\;1}^{+\infty }e^{-\tau_{d}j}\text{cos}(\beta j)L^{j}$.
Case 1.3 $b(L)=\sum_{j\;=\;1}^{+\infty }e^{-\tau_{d}j}\text{cos}(\beta j+\phi )L^{j}$.

Here, τ_*d*_, β and ϕ are constants. We note that there are no oscillations in *b*(*L*) in Case 1.1, and there are oscillations in *b*(*L*) in Cases 1.2 and 1.3 and with a further phase shift in Case 1.3.

For the second class, we do not fix *S*_*xx*_(ω) as a constant, i.e., the time series *X*_*t*_ is no longer white. Instead, we study some special combinations of *a*(*L*) and *b*(*L*). For this class, we cannot derive explicit analytic forms of *F*^(*k*)^_*y*→*x*_. Therefore, we turn to GC values obtained numerically through Equations (14) and (15) to study the relation between the oscillations of *F*^(*k*)^_*y*→*x*_ and the peak frequencies of *S*_*xx*_(ω) and |*S*_*xy*_(ω)|. We will study the following cases in this class:

Case 2.1 $a(L)=\sum_{j\;=\;0}^{+\infty }{\text{e}}^{-\tau_{d}j}\text{cos}(\beta_{1}j)L^{j},\;b(L)=\sum_{j\;=\;1}^{+\infty }{\text{e}}^{-\tau_{d}j}L^{j}$,
Case 2.2 $a(L)=\sum_{j\;=\;0}^{+\infty }{\text{e}}^{-\tau_{d}j}\text{cos}(\beta_{1}j)L^{j},b(L)=\sum_{j\;=\;1}^{+\infty }{\text{e}}^{-\tau_{d}j}\text{cos}(\beta_{2}j)L^{j}$.

Here, τ_*d*_, β_1_, β_2_ are constants. We note that in Cases 2.1 and 2.2 there are oscillations in the time series in *X*_*t*_ with a non-oscillatory *b*(*L*) in Case 2.1 and with an oscillatory *b*(*L*) in Case 2.2.

We now turn to the detailed discussion of the properties of *F*^(*k*)^_*y*→*x*_ in all the listed cases.

*Case 1.1* We consider the case $b(L)=\sum_{j\;=\;1}^{+\infty }{\text{e}}^{-\tau_{d}j}L^{j}$ which has no oscillations in *b*(*L*) and, therefore, the peak frequency of |*S*_*xy*_(ω)| is at 0. We will examine whether *F*^(*k*)^_*y*→*x*_ oscillates with respect to *k*. From Equation (16), Under Approximation I: *C* » *b*^*k*^(ω)*b*^*k*^(ω)^*^, we can derive the following asymptotic result (see Appendix A in Supplementary Material for details)

(18) $$F_{y\to x}^{\left(k\right)}\approx \frac{e^{-2\tau_{d}k}}{C},$$

for which, we can clearly see that GC decreases exponentially with respect to *k*.

**Figure 4:**
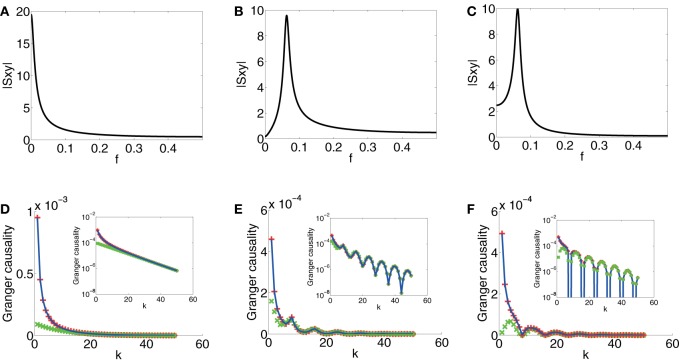
**Contrast between spectra and GC sampling structures for the first class**. Magnitude of *S*_*xy*_ (|*S*_*xy*_|) vs. frequency *f* (*f* = $\frac{\omega }{2\pi }$) (black) for **(A)** Case 1.1. **(B)** Case 1.2. **(C)** Case 1.3. **(D)** Comparison of *F*^(*k*)^_*y* → *x*_ obtained through numerical solution Equation (15) (blue), asymptotic expressions Equation (2) in Supplementary Material (red plus) and Equation (18) (green cross) vs. sampling interval *k* for Case 1.1. **(E)** Comparison of *F*^(*k*)^_*y* → *x*_ obtained through numerical solution Equation (15) (blue), Equation (5) in Supplementary Material (red plus) and Equation (19) (green cross) vs. sampling interval *k* for Case 1.2. **(F)** Comparison of *F*^(*k*)^_*y* → *x*_ obtained through numerical solution Equation (15) (blue), Equation (9) in Supplementary Material (red plus) and Equation (21) (green cross) vs. sampling interval *k* for Case 1.3. Insets are corresponding log-linear plots of GC vs. sampling interval length *k*. The exponential decay is clearly seen in the insets. The parameters are *C* = 10^4^, τ_*d*_ = 0.05, and β = $\frac{\pi }{8}$.

In Figure 4A, which displays spectrum |*S*_*xy*_|, we can observe that the magnitude of *S*_*xy*_ concentrates near 0 which signifies that there are no oscillations induced by the coupling *b*(*L*). In Figure 4D, which displays the GC sampling structure for this case, clearly, there are no oscillations. It can also be seen that GC obtained through the asymptotic result, [Equation (2) in Supplementary Material], agrees very well with the numerically obtained GC for all *k*'s and the asymptotic formula Equation (18) approximates GC rather well when *k* is large. Therefore, we can conclude that if there are no oscillations in the coupling *b*(*L*), as signified in the peak frequency of |*S*_*xy*_| near 0 and if *S*_*xx*_ and *S*_*yy*_ are constant (i.e., both are white), then *F*^(*k*)^_*y*→*x*_ does not oscillate with respect to *k*.

*Case 1.2* From Case 1.1 in Model I, no oscillations in *b*(*L*) implies no oscillations in the GC sampling structure. Next, we consider $b(L)=\sum_{j\;=\;1}^{+\infty }{\text{e}}^{-\tau_{d}j}\text{cos}(\beta j)L^{j}$ to examine whether oscillations in the coupling *b*(*L*) may induce oscillations in the GC sampling structure.

Under Approximation II, i.e., large τ and *k*, we can obtain the following asymptotic expression through Equation (16) (see Appendix A in Supplementary Material for details)

(19) $$F_{y\to x}^{\left(k\right)}\approx \frac{1}{C}\left(e^{-4\tau_{d}k}+e^{-2\tau_{d}k}{\text{cos}}^{2}\beta k\right),$$

from which we can observe that there are oscillations in the GC sampling structure.

In Figure 4B, the peak of |*S*_*xy*_(ω)| is located approximately at *f* = $\frac{1}{16}$, which faithfully reflects the oscillation frequency *f*_0_ (*f*_0_ = $\frac{\beta }{2\pi }$ = $\frac{1}{16}$) of *b*(*L*). In Figure 4E, the oscillation frequency *f*′_0_ of *F*^(*k*)^_*y*→*x*_ is approximately $\frac{1}{8}$, which is twice of *f*_0_. In addition, the curve obtained by using Approximation I [Equation (5) in Supplementary Material] overlaps with the numerically computed curve, and for large *k*, the curve obtained by using Approximation II, Equation (19), fits well with the numerically computed curve. Therefore, from Equation (19), we can conclude that oscillations at frequency *f*_0_ in the coupling *b*(*L*), manifested as the peak frequency *f* = *f*_0_ = $\frac{\beta }{2\pi }$ in |*S*_*xy*_|, implies double frequency oscillations (*f*′_0_ = 2*f*_0_ = $\frac{\beta }{\pi }$) in the GC sampling structure.

*Case 1.3* By the study of Case 1.2, we can further ask how the phase of oscillations in *b*(*L*) regulates the oscillations in the GC sampling structure. We consider oscillations with phase ϕ in *b*(*L*), that is, $b(L)=\sum_{j\;=\;1}^{+\infty }e^{-\tau_{d}j}\text{cos}(\beta j+\phi )L^{j}$. Under Approximation II, we obtain following asymptotic form through Equation (16) (see Appendix A in Supplementary Material for details)

(20) $$F_{y\to x}^{\left(k\right)}\approx \frac{1}{C}e^{-2\tau_{d}k}{\text{cos}}^{2}(\beta k+\phi ).$$

For a special case, when ϕ = − $\frac{\pi }{2}$, Equation (20) becomes

(21) $$F_{y\to x}^{\left(k\right)}\approx \frac{1}{C}e^{-2\tau_{d}k}{\text{sin}}^{2}\beta k.$$

In Figure 4C, which depicts the spectrum of *S*_*xy*_, the peak frequency of |*S*_*xy*_| is the same as the peak frequency *f* = $\frac{1}{16}$ in Figure 4B, and this reflects the same frequency of the oscillations in the coupling term *b*(*L*). In Figure 4F, the oscillation frequency *f*′_0_ of *F*^(*k*)^_*y*→*x*_ is again twice of the peak frequency of |*S*_*xy*_| [as can be seen in Equation (21)]. In addition, the GC sampling structure via the approximation of Equation (9) in Supplementary Material is in excellent agreement with numerically solved GC sampling structure for all *k*'s. For large *k*, the asymptotic expression of Equation (21) fits well with numerically solved GC sampling structure. However, in contrast to Case 1.2, there is a shift in *k* for the points at which *F*^(*k*)^_*y*→*x*_ attains minimum (or maximum). Therefore, from Equation (20), we can conclude that the phase factor ϕ in *b*(*L*) does not affect the frequency of *F*^(*k*)^_*y*→*x*_ but it regulates the phase of the oscillations, i.e., it changes the locations of the extrema of *F*^(*k*)^_*y*→*x*_ depending on ϕ. We also note that, in the present case, the minimum value of *F*^(*k*)^_*y*→*x*_ in oscillations approaches 0.

*Case 2.1 and Case 2.2* For the first class we studied above, we have *S*^(*k*)^_*xx*_(ω) ≡ *C* and *S*^(*k*)^_*yy*_(ω) ≡ 1, which do not contribute to the variations of *F*^(*k*)^_*y*→*x*_. Then, we obtain a direct relation between the cross-spectrum *S*_*xy*_(ω) and the GC sampling structure. However, because *S*_*xx*_(ω) often has peaks at some frequencies especially for time series in neural systems, we further examine the role of *S*_*xx*_(ω) [or *S*_*yy*_(ω)] in the oscillations of the GC sampling structure. Therefore, we turn to the study of the second class. In order to examine how the oscillation of the causality depends on the oscillations of *a*(*L*) and *b*(*L*), we examine the large μ limit, and thus, the location of frequency in the peak of *S*_*xx*_(ω) is determined by *a*(*L*), whereas the peak of *S*_*xy*_(ω) is determined by *b*(*L*).

**Figure 5:**
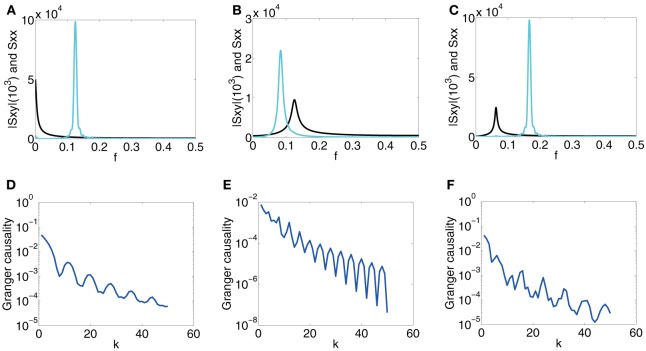
**Contrast between spectra and the GC sampling structure for the second class**. The top row of the panel is spectra *S*_*xx*_ (cyan) and |*S*_*xy*_| (black), which is three orders of magnitude smaller than that of *S*_*xx*_ and is magnified by a factor 10^3^, vs. frequency *f*, **(A)** Case 2.1 with parameters β_1_ = $\frac{\pi }{4}$, τ_d_ = 0.02, μ = 200. **(B)** Case 2.2 with parameters β_1_ = $\frac{\pi }{6}$, β_2_ = $\frac{\pi }{4}$, τ_*d*_ = 0.05, μ = 200. **(C)** Case 2.2 with parameters β_1_ = $\frac{\pi }{3}$, β_2_ = $\frac{\pi }{8}$, τ_*d*_ = 0.02, μ = 200. **(D–F)** the corresponding log-linear plots of *F*^(*k*)^_*y* → *x*_ obtained numerically (blue) with respect to sampling interval length *k*.

First, for $a(L)=\sum_{j\;=\;0}^{+\infty }{\text{e}}^{-\tau_{d}j}\text{cos}(\beta_{1}j)L^{j}$, $b(L)=\sum_{j\;=\;1}^{+\infty }{\text{e}}^{-\tau_{d}j}L^{j}$, from which there are no oscillations in the coupling *b*(*L*), we study the behavior of *F*^(*k*)^_*y*→*x*_ to look for the relation between the oscillations in *F*^(*k*)^_*y*→*x*_ and oscillations in *a*(*L*). In Figure 5A, which displays spectra of *S*_*xx*_ and |*S*_*xy*_|, the peak of *S*_*xx*_ is located approximately at *f* = $\frac{1}{8}$, which reflects the oscillation frequency in *a*(*L*) and |*S*_*xy*_| concentrates near 0, which reflects the non-oscillatory nature of *b*(*L*). In Figure 5D, which displays the corresponding GC sampling structure, the oscillation frequency *f*_0_ in the GC sampling structure is approximately $\frac{1}{8}$, which equals to the peak frequency of *S*_*xx*_ as well as the oscillation frequency in *a*(*L*). In this case, we conclude that *S*_*xx*_ with a peak at *f*_0_ can induce oscillations with frequency *f*_0_ in the GC sampling structure.

Second, for the case of $a(L)=\sum_{j\;=\;0}^{+\infty }{\text{e}}^{-\tau_{d}j}\text{cos}(\beta_{1}j)L^{j}$, and $b(L)=\sum_{j\;=\;1}^{+\infty }{\text{e}}^{-\tau_{d}j}\text{cos}(\beta_{2}j)L^{j}$, in which both have oscillations with frequency β_1_ and β_2_, respectively, we investigate the interactions of different oscillations in *a*(*L*) and *b*(*L*) in inducing the oscillations in *F*^(*k*)^_*y*→*x*_. From the studies above, one may suspect that the oscillations in *F*^(*k*)^_*y*→*x*_ should be a combination of frequency $\frac{\beta_{1}}{2\pi }$ and $\frac{\beta_{2}}{\pi }$, implying that we may encounter complicated oscillation structures for *F*^(*k*)^_*y*→*x*_ with frequency consisting of $\frac{\beta_{1}}{2\pi }$ and $\frac{\beta_{2}}{\pi }$. Shown in Figures 5E,F are such cases. In Figure 5B, the peak frequencies of *S*_*xx*_ and |*S*_*xy*_| meet the oscillation frequency of *a*(*L*) ($\frac{\beta_{1}}{2\pi }$ = $\frac{1}{12}$) and *b*(*L*) ($\frac{\beta_{2}}{2\pi }$ = $\frac{1}{8}$), respectively. The GC sampling structure is shown in Figure 5E, where *F*^(*k*)^_*x*,*y*_ oscillates at frequency $\frac{1}{4}$ which is twice of $\frac{\beta_{2}}{2\pi }$ and is three times of $\frac{\beta_{1}}{2\pi }$. In Figure 5C, the peak frequencies of *S*_*xx*_ and |*S*_*xy*_| meet the oscillation frequency of *a*(*L*) ($\frac{\beta_{1}}{2\pi }$ = $\frac{1}{6}$) and *b*(*L*) ($\frac{\beta_{2}}{2\pi }$ = $\frac{1}{16}$), respectively. The GC sampling structure is displayed in Figure 5F, in which the oscillation features are complicated, making it difficult to discern simple frequencies. Therefore, the results in Figure 5 confirm that both oscillations of *a*(*L*) and *b*(*L*) can contribute to the oscillations in the GC sampling structure whose oscillation frequency can be a combination of those in *a*(*L*) and *b*(*L*).

#### 3.2.3. Idealized model II

In Model I, there is no oscillation in the white noise time series *Y*_*t*_. For Case 1, the oscillations in the GC sampling structure are related to the peak in *S*_*xy*_, whereas, for Case 2, the GC oscillations are related to the interplay between peaks in *S*_*xy*_ and *S*_*xx*_. However, in the reconstruction of the topology of the simple two-neuron network, as shown in Figure 2C, there are peaks at approximately the same frequency in *S*_*xx*_, |*S*_*xy*_|, *S*_*yy*_, and this frequency is half of the oscillation frequency in the GC sampling structure. We consider the following linear model that possesses the same properties as those in the I&F network case, i.e., *S*_*xx*_, |*S*_*xy*_|, and *S*_*yy*_ all possess a peak at the same frequency:

(22) $$\{\begin{array}{ll} X_{t} & =a(L)\epsilon_{t}+b(L)\eta_{t},\\ Y_{t} & =c(L)\eta_{t}, \end{array}$$

where the covariance matrix Σ for the white noise time series ϵ_*t*_, η_*t*_ is $\Sigma =\left[\begin{array}{cc} 1 & 0\\ 0 & 1 \end{array}\right]$. Then, *S*_*xx*_ = *a*(ω)a^*^(ω) + *b*(ω)b^*^(ω), *S*_*yy*_ = *c*(ω)c^*^(ω), and *S*_*xy*_ = *b*(ω)c^*^(ω). In this case, we set $a(L)=\;\sum_{j\;=\;0}^{+\infty }{\text{e}}^{-\tau_{d}j}\text{cos}(\beta j)L^{j}$, $b(L)=\gamma \sum_{j\;=\;1}^{+\infty }{\text{e}}^{-\tau_{d}j}\text{cos}(\beta j+\phi )L^{j}$, $c(L)=\sum_{j\;=\;0}^{+\infty }{\text{e}}^{-\tau_{d}j}\text{cos}(\beta j)L^{j}$, where τ_*d*_, β, γ, ϕ are constants. Note that this model is different from Idealized Model I in that we no longer have *F*^(*k*)^_*y*→*x*_ ≡ *F*^(*k*)^_*x*,*y*_ and *F*^(*k*)^_*x*→*y*_ ≡ *F*^(*k*)^_*x*·*y*_ ≡ 0.

**Figure 6:**
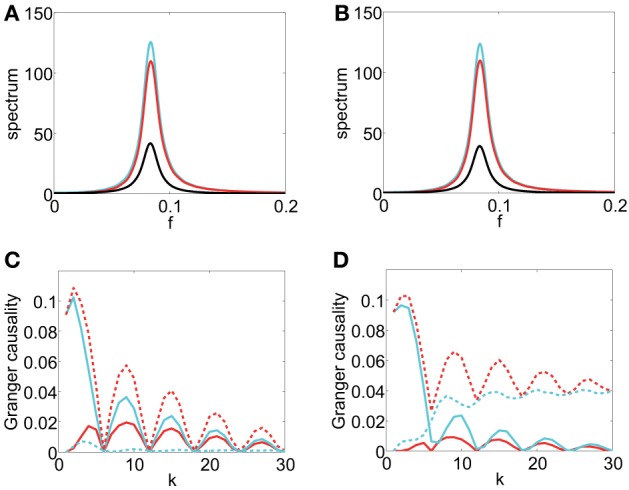
**Contrast between spectra and the GC sampling structure obtained numerically for Model II**. *S*_*xx*_ (cyan), |*S*_*xy*_| (black), *S*_*yy*_ (red) with parameters τ_*d*_ = 0.05, β = $\frac{\pi }{6}$, γ = 0.4, **(A)** ϕ = $\frac{\pi }{2}$, **(B)** ϕ = $\frac{\pi }{4}$. *F*_*x* → *y*_ (red), *F*_*y* → *x*_ (cyan), *F*_*x*,*y*_ (red dash), *F*_*x*·*y*_ (cyan dash) with respect to sampling interval length *k* with the parameters **(C)** same as the parameters in **(A)**. **(D)** same as the parameters in **(B)**.

From the spectrum plots shown in Figures 6A,B, it can be seen that the peak frequencies of all spectra are approximately $\frac{1}{12}$. Figures 6C,D display the corresponding GC sampling structure. We can observe that the oscillation frequencies in the GC sampling structure are approximately $\frac{1}{6}$, which is again twice of the peak frequency in Figures 6A,B. Therefore, for both the linear model (22) and the I&F networks, there is the same relation between the frequency in the GC sampling structure and that of the peaks in the spectra as those shown in Figure 2. We note that the phase difference ϕ between *b*(*L*) and *a*(*L*) [or *c*(*L*)] may lead to a rather different behavior of the instantaneous Granger causality, *F*_*x*·*y*_, as demonstrated in the two cases shown in Figures 6C,D (ϕ = $\frac{\pi }{2}$ in Figure 6C and ϕ = $\frac{\pi }{4}$ in Figure 6D). Note that, by construction, there is no causal influence from *X*_*t*_ to *Y*_*t*_ in Equation (22). However, *F*_*x*→*y*_ in Figures 6C,D show significantly non-zero values for ranges of *k*. Clearly, these GC values are spurious and potentially give rise to erroneous causal inferences even in this linear setting. As pointed out previously, this spurious GC phenomenon also occurs in the I&F neuronal networks (see Figure 2A).

In summary, from the results above, we see that the oscillations in the GC sampling structure are related to the oscillations in the original time series and in the coupling of the time series regardless of their linear or non-linear nature. For special cases, there is a doubling frequency in the GC oscillations in comparison with the peak frequency in spectra. However, in general, there may not be a simple relation between these oscillation frequencies. Nevertheless, we can infer that, in general, all oscillations manifested as peaks of *S*_*xx*_, |*S*_*xy*_| and *S*_*yy*_ may give rise to oscillations in the GC sampling structure.

### 3.3. Vanishing GC as τ→0

We have already observed in Figure 3 that the GC value vanishes as the sampling interval length τ tends to 0 for GC obtained from both voltage and spike train time series. Moreover, we note that Figure 3 also exhibits that the GC value approaches 0 in a manner almost linearly proportional to the sampling interval length τ as τ → 0. As mentioned before, this sampling effect gives rise to the paradox that GC analysis seems to be less reliable as more information is incorporated as τ → 0. In the following, we will study the mechanisms of such effect and address the question of extracting reliable causal interaction as τ → 0 in order to resolve this paradox.

Because GC can be analyzed through the spectra, we first study the limit of the spectral matrix **S**^(τ)^(ω), which is the spectrum of the time series with sampling interval length τ. Suppose that we perform a uniform sampling of a bivariate continuous-time stationary process *X*_*t*_ and *Y*_*t*_ with sampling interval length τ to obtain *X*_*n*τ_, *Y*_*n*τ_, where *n* is an integer. Using the Wiener-Khinchin theorem, through the relation between the time correlation of discrete time series and that of the original continuous signal, we can obtain the relation between the corresponding spectral matrix **S**^(τ)^(ω) and the power spectral density **P**(*f*) (ω = 2πτ*f*) of the continuous-time processes *X*_*t*_ and *Y*_*t*_ as follows (see Appendix B in Supplementary Material for details):

(23) $$\tau \left[\begin{array}{cc} S_{xx}^{\left(\tau \right)}(\omega ) & S_{xy}^{\left(\tau \right)}(\omega )\\ S_{yx}^{\left(\tau \right)}(\omega ) & S_{yy}^{\left(\tau \right)}(\omega ) \end{array}\right]\to \left[\begin{array}{cc} P_{xx}(f) & P_{xy}(f)\\ P_{yx}(f) & P_{yy}(f) \end{array}\right]$$

as τ → 0. Therefore, **S**^(τ)^(ω) is scaled as $\frac{1}{\tau }$ as τ → 0.

Using Equation (12), total Granger causality *F*^(τ)^_*x*,*y*_ with sampling interval length τ can be directly computed using each component of spectral matrix **S**^(τ)^(ω), which is

(24) $$F_{x,y}^{\left(\tau \right)}=-\frac{1}{2\pi }\int_{-\pi }^{\pi }\ln\left(1-\frac{S_{xy}^{\left(\tau \right)}(\omega )S_{yx}^{\left(\tau \right)}(\omega )}{S_{xx}^{\left(\tau \right)}(\omega )S_{yy}^{\left(\tau \right)}(\omega )}\right)\text{d}\omega .$$

Taking the limit of τ → 0 and using Equation (23), we obtain

(25) $$\frac{1}{\tau }F_{x,y}^{\left(\tau \right)}\to -\int_{-\infty }^{+\infty }\ln\left[1-C(f)\right]\text{d}f$$

as τ → 0, where $C(f)=\frac{P_{xy}(f)P_{yx}(f)}{P_{xx}(f)P_{yy}(f)}$ is the coherence of continuous-time processes *X*_*t*_, *Y*_*t*_.

Because a spectral matrix possesses the property of factorization (Wilson, 1972), i.e., **S**(ω) = **A**(*e*^*i*ω^)A^*^(*e*^*i*ω^), where ^*^ denotes matrix adjoint, the factorization of the spectral matrix gives rise to the decomposition **S**^(τ)^(ω) = **H**^(τ)^(ω)Σ^(τ)^**H**^(τ)^(ω)^*^ of spectral matrix **S**^(τ)^(ω), where **H**^(τ)^(ω) = **A**^(τ)^(*e*^*i*ω^)**A**^(τ)^(0)^−1^, and the covariance matrix Σ^(τ)^ = **A**^(τ)^(0)**A**^(τ)^(0)^*^, $H=\left[\begin{array}{cc} H_{xx} & H_{xy}\\ H_{yx} & H_{yy} \end{array}\right]$, $\Sigma =\left[\begin{array}{cc} \Sigma_{2} & {ϒ}_{2}\\ {ϒ}_{2} & \Gamma_{2} \end{array}\right]$ (see Appendix B in Supplementary Material for details). Defining $\hat{H}(f)=\lim_{\tau \;\to \;0}\tau H^{\left(\tau \right)}(2\pi \tau f)$, $\hat{\Sigma }=\lim_{\tau \;\to \;0}\frac{1}{\tau }\Sigma^{\left(\tau \right)}$, we obtain the limit expressions for *F*^(τ)^_*x*→*y*_, *F*^(τ)^_*y*→*x*_ and *F*^(τ)^_*x*·*y*_, which are

(26) $$\frac{1}{\tau }F_{y\to x}^{\left(\tau \right)}\;\to \;\int_{-\infty }^{+\infty }\ln\frac{P_{xx}(f)}{{\hat{H}}_{xx}(f){\hat{\Sigma }}_{2}{\hat{H}}_{xx}^{*}(f)}\text{d}f,$$

(27) $$\frac{1}{\tau }F_{x\to y}^{\left(\tau \right)}\to \int_{-\infty }^{+\infty }\ln\frac{P_{yy}(f)}{{\hat{H}}_{yy}(f){\hat{\Gamma }}_{2}{\hat{H}}_{yy}^{*}(f)}\text{d}f,$$

(28) $$\frac{1}{\tau }F_{x\cdot y}^{\left(\tau \right)}\to \text{ ​}-\text{ ​}\int_{-\infty }^{+\infty }\ln\text{​}\left\{\text{​}\frac{P_{xx}(f)P_{yy}(f)}{\left[{\hat{H}}_{xx}(f){\hat{\Sigma }}_{2}{\hat{H}}_{xx}^{*}(f)\right]\left[{\hat{H}}_{yy}(f){\hat{\Gamma }}_{2}{\hat{H}}_{yy}^{*}(f)\right]}-\text{​}1\text{​}\right\}\text{d}f\text{​},\;\;\;$$

as τ → 0.

Then, if $\int_{-\infty }^{+\infty }\ln\left[1-C(f)\right]\text{d}f$ is finite, we can easily show that $\frac{1}{\tau }$*F*^(τ)^_*x*,*y*_, $\frac{1}{\tau }$*F*^(τ)^_*x*→*y*_, $\frac{1}{\tau }$*F*^(τ)^_*y*→*x*_ and $\frac{1}{\tau }$*F*^(τ)^_*x*·*y*_ all approach finite values in the limit of τ → 0. Therefore, the Granger causality is linearly proportional to the sampling interval length τ for small τ. Hence, vanishing *F*^(τ)^_*x*,*y*_, *F*^(τ)^_*x*→*y*_, *F*^(τ)^_*y*→*x*_ and *F*^(τ)^_*x*·*y*_ as τ → 0. The corresponding limits are related to the intrinsic properties of the continuous-time processes.

## 4. Discussion and conclusion

### 4.1. Selection of sampling intervals in the application of GC

From our results above, it becomes evident that one has to be careful in interpreting causal inference using the GC analysis. For sufficiently large sampling interval length, the GC in general decays exponentially to zero because much of information over dominant frequencies is lost and the GC value becomes small and unreliable for causal inference. When there is causal flow, to avoid a possible vanishing GC value due to zeroes in the oscillations in the GC structure, one should use a range of sampling interval lengths to obtain discrete time series to ascertain the general features of the GC sampling structure, such as Figure 2, so as to avoid using accidental vanishing GC values for causal inference and to obtain a reliable interpretation of causality. When there is no causal flow, spurious causality may also arise (e.g., Figures 2, 6) when the sampling interval length is large because the loss of high frequency information for self prediction of one time series can possibly be compensated by the other time series.

In order to preserve the high frequency information, one would use very fine sampling intervals to sample a continuous-time process. However, as we have pointed out above that the GC values exhibit a linear relation with τ as the sampling interval vanishes, thus, again leading to seemingly unreliable inference of causality. We can circumvent this difficulty by using the following procedure.

**Figure 7:**
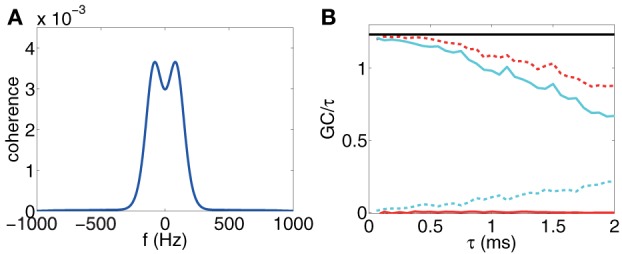
**(A)** Coherence vs. frequency *f*. (b) $\frac{1}{\tau }$*F*^(τ)^_*x* → *y*_ (red), $\frac{1}{\tau }$*F*^(τ)^_*y* → *x*_ (cyan), $\frac{1}{\tau }$*F*^(τ)^_*x*,*y*_ (red dash), $\frac{1}{\tau }$*F*^(τ)^_*x* · *y*_ (cyan dash) for voltage time series and $\frac{1}{\tau }$*F*_*x*,*y*_ computed through Equation (25) (black horizontal line) vs. sampling interval length τ. The time series are generated by the I&F network whose topology is shown in Figure 1A with parameters ν = 1 ms^−1^, *s* = 0.02, λ = 0.0177. Note that we have subtracted the estimation bias of GC from the estimate of GC values for **(B)**. The procedure of removing biases is described in Appendix C in Supplementary Material.

First, for a range of small τ's, we ascertain the linear range of the GC value as a function of τ, then we plot the ratio of the GC value to the corresponding sampling interval length τ to extract its limiting value as τ → 0, e.g., by Equation (25), Figure 7 shows such an example for time series sampled from the I&F network dynamics. It is clear that the directed couplings of the neuronal network dynamics are correctly inferred by the clearly non-zero ratio for the presence of the synaptic coupling and a vanishing ratio for the absence of synaptic coupling. According to asymptotic distributions of GCs, the GC computed from time series is a biased estimate, e.g., the bias is $\frac{p}{n}$ for *F*_*x* → *y*_, where *p* is the regression order, *n* is the length of time series. To plot the ratio of GC to τ, we used the numerical computed GC with the bias subtracted (see Appendix C in Supplementary Material for details).

In applications, we may wonder how we determine this linear range and whether this linear range is sufficiently large for the GC extraction. Here, we present an estimation using Equation (25). The idea is that if $\int_{-\frac{1}{2\tau_{0}}}^{\frac{1}{2\tau_{0}}}\ln\left[1-C(f)\right]\text{d}f$ is a good approximation of $\int_{-\infty }^{+\infty }\ln\left[1-C(f)\right]\text{d}f$ for some τ_0_, which implies that the causal information with frequency higher than $\frac{1}{2\tau_{0}}$ can be ignored, then, τ_0_ is inside the linear range for small GCs. We can determine such range by inspecting directly the figure of *C*(*f*) and choose a proper cut-off frequency *f*_0_ such that *C*(*f*) is sufficiently small, thus can be ignored when |*f*| > *f*_0_. We take the voltage time series of a two-neuron I&F network as an example. Figure 7A displays the coherence function *C*(*f*). It can be seen from the figure that we can choose the cut-off frequency *f*_0_ = 500 Hz, above which the coherence almost vanishes. Then, we can conclude that the linear range for GC is from 0 ms to ~1 ms. Figure 7B demonstrates the validity of our estimate where $\frac{1}{\tau }$*F*^(τ)^_*x*,*y*_ is approximately a constant over the range from 0 ms to ~1 ms of the sampling interval. Therefore, it is sufficient to use voltage time series sampled with time interval 0.5 ms or less to extract good approximations of the limiting GC-to-τ ratios. We note in passing that, in the Method section, we used τ = 0.5 ms to evaluate the GC values for the network reconstruction.

### 4.2. Conclusions

In this work, we have discussed general features of the GC sampling structure and their strong implications for causal inference by using the GC analysis. We have also discussed a general approach that can overcome these artifacts arising from sampling interval lengths to obtain reliable GC values. We note that these issues arise when we sample continuous-time processes to obtain discrete time series for the GC analysis. Therefore, the issues should be examined regardless of whether one uses parametric or non-parametric methods for the GC estimation. Furthermore, the general strategies of overcoming these sampling issues are not limited to the bivariate time series with unidirectional connection as discussed in this work. They are also applicable to the GC analysis of bivariate time series with bidirectional connections (shown in Supplementary Figure 2) as well as multivariate time series with any general connectivity structure (Zhou et al., 2013b, 2014).

## Author contributions

Conceived and designed the research: Douglas Zhou, Yaoyu Zhang, Yanyang Xiao, and David Cai. Performed experiments and analyzed data: Douglas Zhou, Yaoyu Zhang, Yanyang Xiao, and David Cai. Wrote the paper: Douglas Zhou, Yaoyu Zhang, Yanyang Xiao, and David Cai.

### Conflict of interest statement

The authors declare that the research was conducted in the absence of any commercial or financial relationships that could be construed as a potential conflict of interest.
