# Supplementary material for: Analysis of sampling artifacts on the Granger causality analysis for topology extraction of neuronal dynamics
Source: Front Comput Neurosci. 2014 Jul 30;8:75. doi: 10.3389/fncom.2014.00075 (PMC4115622; doi:10.3389/fncom.2014.00075)
Supplement: Supplementary file 1 [file Presentation1.PDF]

Frontiers in Computational Neuroscience

Research Article
7 July 2014

Supplementary Material: Analysis of Sampling Artifacts on the Granger Causality Analysis for Topology Extraction of Neuronal Dynamics

Douglas Zhou [1*], **Yaoyu Zhang** [1], **Yanyang Xiao** [1], **David Cai** [1,2,3*]

[1] *Department of Mathematics, MOE-LSC and Institute of Natural Sciences, Shanghai Jiao Tong University, Shanghai, China*
[2] *Courant Institute of Mathematical Sciences and Center for Neural Sciences, New York University, New York, NY, USA*
[3] *NYUAD Institute, New York University Abu Dhabi, Abu Dhabi, UAE*

Correspondence*:
Douglas Zhou
Department of Mathematics, MOE-LSC, Institute of Natural Sciences, Shanghai Jiao Tong University, 800 Dongchuan Road, Shanghai, 200240, China,
zdz@sjtu.edu.cn

David Cai
Department of Mathematics, MOE-LSC, Institute of Natural Sciences, Shanghai Jiao Tong University, 800 Dongchuan Road, Shanghai, 200240, China,
cai@cims.nyu.edu

1 SUPPLEMENTARY DATA

1.1 APPENDIX A

In this section, we describe in detail certain results about sampling in the main text.

1.1.1 Spectrum with sampling interval k Here we show that $S^{(k)}(\omega)$ is given by Eq. (14) in the main text in which $S(\omega)$ is the spectrum for the original time series $\{X_0, X_1, X_2, \cdots\}$, k is the sampling interval length, which is a positive integer, for discrete time series, and $S^{(k)}(\omega)$ is the spectrum for time series $\{X_0, X_k, X_{2k}, \cdots\}$. By the Wiener-Khinchin theorem, the spectrum of a time series can be written as the Fourier transform of the covariance series $\mathrm{cov}(n)$, *i.e.*, $\mathrm{cov}(n) \equiv \mathrm{E}(X_t, X_{t+n})$, n is an integer, *i.e.*

$$S(\omega) = \sum_{n=-\infty}^{+\infty} \mathrm{cov}(n) e^{in\omega},$$

and

$$S^{(k)}(\omega) = \sum_{n=-\infty}^{+\infty} \mathrm{cov}(nk) e^{in\omega}.$$

Then

$$\begin{aligned}\frac{1}{k}\sum_{j=0}^{k-1} S(\frac{\omega}{k}+\frac{2\pi j}{k}) &= \frac{1}{k}\sum_{j=0}^{k-1}\sum_{n=-\infty}^{+\infty} \mathrm{cov}(n) e^{in(\frac{\omega}{k}+\frac{2\pi j}{k})}, \\ &= \frac{1}{k}\sum_{n=-\infty}^{+\infty}\left(\mathrm{cov}(n) e^{i\frac{n}{k}\omega}\sum_{j=0}^{k-1} e^{i\frac{2\pi n}{k}j}\right).\end{aligned}$$

For $\sum_{j=0}^{k-1} e^{i\frac{2\pi n}{k}j}$, only when $n = mk$ (m is an integer), it does not vanish and it is equal to k. Thus,

$$\begin{aligned}\frac{1}{k}\sum_{j=0}^{k-1} S(\frac{\omega}{k}+\frac{2\pi j}{k}) &= \frac{1}{k}\sum_{m=-\infty}^{+\infty}\left(\mathrm{cov}(mk) e^{i\frac{mk}{k}\omega} k\right), \\ &= \sum_{m=-\infty}^{+\infty} \mathrm{cov}(mk) e^{im\omega}, \\ &= S^{(k)}(\omega),\end{aligned}$$

which is Eq. (14) in the main text as we desire to show.

1.1.2 Special bivariate time series yielding $F^{(k)}_{x\to y} \equiv F^{(k)}_{x\cdot y} \equiv 0$ Here we briefly illustrate that for discrete time series X_t and Y_t, if Y_t, t is an integer, is a white noise series and $\mathrm{cov}(Y_t, X_{t-i}) = 0$ for any positive integer $i > 0$, then $F^{(k)}_{x\to y} \equiv 0$. In particular, when $\mathrm{cov}(Y_t, X_{t-i}) = 0$ for any integer $i \geqslant 0$, $F^{(k)}_{x\to y} \equiv F^{(k)}_{x\cdot y} \equiv 0$.

Note that the time series with sampling interval k for X_t, Y_t, t is an integer, are X_{tk} and Y_{tk}. Since Y_{tk} is a white noise series, the auto-regression residual series is Y_{tk} itself. Thus, $\Gamma^{(k)}_1 = \mathrm{var}(Y_{tk}) = \mathrm{var}(Y_t)$. Since Y_{tk} is uncorrelated with the set $X_{(t-i)k}$, $Y_{(t-i)k}$ (integer $i > 0$), the joint regression residual series is also Y_{tk} itself. Therefore, $\Gamma^{(k)}_2 = \Gamma^{(k)}_1 = \mathrm{var}(Y_t)$. By definition (6) in the main text, $F^{(k)}_{x\to y} = \ln \frac{\Gamma^{(k)}_1}{\Gamma^{(k)}_2} = 0$.

When $\mathrm{cov}(Y_t, X_{t-i}) = 0$ for $i \geqslant 0$, Y_{tk} is uncorrelated with $X_{(t-i)k}$, $i \geqslant 0$ and $Y_{(t-j)k}$, $j > 0$, which implies that $\Upsilon^{(\mathrm{k})}_2 = \mathrm{E}(\epsilon_{2(tk)}, Y_{2(tk)}) = 0$ (otherwise Y_{tk} is correlated with $X_{(t-i)k}$, $i \geqslant 0$). Then, by definition (7) in the main text, we obtain $F^{(k)}_{x\cdot y} = \ln \frac{\Gamma^{(k)}_2 \Sigma^{(k)}_2}{|\boldsymbol{\Sigma}^{(k)}|} = 0$.

1.1.3 Computation of $F^{(k)}_{y\to x}$ in Case 1.1 We consider the case $b(L) = \sum_{j=1}^{+\infty} e^{-\tau_d j} L^j$, which has no oscillations in $b(L)$. From Eq. (12) in the main text, we have

$$F^{(k)}_{y\to x} = \ln C - \frac{1}{2\pi}\int_{-\pi}^{\pi} \ln\left[C - b^{(k)}(\omega) b^{(k)}(\omega)^*\right] \mathrm{d}\omega, \tag{1}$$

where $C = a(\omega)a^*(\omega)\mu + b(\omega)b^*(\omega)$, and $b^{(k)}(\omega) = \sum_{j=1}^{+\infty} e^{-\tau_d kj} e^{-i\omega j}$, which can be simplified as

$$b^{(k)}(\omega) = \frac{e^{-\tau_d k} e^{-i\omega}}{1 - e^{-\tau_d k} e^{-i\omega}}.$$

For $C \geqslant \frac{1}{(e^{\tau_d}-1)^2}$, we obtain

$$F^{(k)}_{y\to x} = -\ln\frac{1}{2}\left(1+(1-\frac{1}{C})e^{-2\tau_d k}+\sqrt{\left(1+(1-\frac{1}{C})e^{-2\tau_d k}\right)^2-4e^{-2\tau_d k}}\right). \quad (2)$$

Under Approximation I: $C \gg b^{(k)}(\omega)b^{(k)}(\omega)^*$, we have

$$F^{(k)}_{y\to x} \approx \frac{e^{-2\tau_d k}}{C}, \quad (3)$$

which is Eq. (18) in the main text.

1.1.4 Computation of $F^{(k)}_{y\to x}$ in Case 1.2 We consider $b(L)=\sum_{j=1}^{+\infty} e^{-\tau_d j}\cos(\beta j)L^j$ to examine whether oscillations in the coupling $b(L)$ may induce oscillations in the GC sampling structure.

For this case, we have

$$\begin{aligned} b^{(k)}(\omega) &= \sum_{j=1}^{+\infty} e^{-\tau_d kj}\cos(\beta kj)e^{-i\omega j}, \\ &= e^{-\tau_d k}e^{-i\omega}\frac{-e^{-\tau_d k}e^{-i\omega}+cos\beta k}{(1-e^{-\tau_d k}e^{-i\omega}e^{i\beta k})(1-e^{-\tau_d k}e^{-i\omega}e^{-i\beta k})}. \end{aligned} \quad (4)$$

Under Approximation I: $C \gg b^{(k)}(\omega)b^{(k)}(\omega)^*$, we have

$$F^{(k)}_{y\to x} = \frac{e^{-2\tau_d k}(1-3e^{-2\tau_d k})\cos^2\beta k+e^{-4\tau_d k}+e^{-6\tau_d k}}{C(1-e^{-2\tau_d k})(1+e^{-2\tau_d k}-2e^{-\tau_d k}\cos\beta k)(1+e^{-2\tau_d k}+2e^{-\tau_d k}\cos\beta k)}. \quad (5)$$

Under Approximation II, *i.e.,* large τ_d and k, we can obtain a simplified approximate expression of Eq. (5)

$$F^{(k)}_{y\to x} \approx \frac{1}{C}(e^{-4\tau_d k}+e^{-2\tau_d k}\cos^2\beta k), \quad (6)$$

which is Eq. (19) in the main text.

1.1.5 Computation of $F^{(k)}_{y\to x}$ in Case 1.3 We consider oscillations with phase ϕ in $b(L)$, that is, $b(L)=\sum_{j=1}^{+\infty} e^{-\tau_d j}\cos(\beta j+\phi)L^j$. Under Approximation I, we have

$$\begin{aligned} F^{(k)}_{y\to x} &\approx \frac{1}{2\pi}\int_{-\pi}^{\pi}\frac{b^{(k)}(\omega)b^{(k)}(\omega)^*}{C}\mathrm{d}\omega, \\ &= \frac{e^{-2\tau_d k}\cos^2(\beta k+\phi)-\frac{1}{2}e^{-4\tau_d k}(2\cos 2\beta k+\cos 2\phi+\cos 2(\beta k+\phi))+e^{-6\tau_d k}\cos^2\phi}{C(1+e^{-2\tau_d k}-2e^{-\tau_d k}\cos\beta k)(1+e^{-2\tau_d k}+2e^{-\tau_d k}\cos\beta k)(1-e^{-2\tau_d k})}, \end{aligned} \quad (7)$$

where the dominant oscillation term is $e^{-2\tau_d k}\cos^2(\beta k+\phi)$. Under this approximation, the following approximation is obtained

$$F^{(k)}_{y\to x} \approx \frac{1}{C}e^{-2\tau_d k}\cos^2(\beta k+\phi), \quad (8)$$

which is Eq. (20) in the main text. For a special case, when $\phi = -\frac{\pi}{2}$, from Eq. (7), we obtain

$$F^{(k)}_{y\to x} \quad \approx \quad \frac{e^{-2\tau_d k}(1+e^{-2\tau_d k})\sin^2\beta k}{C(1+e^{-2\tau_d k}-2e^{-\tau_d k}\cos\beta k)(1+e^{-2\tau_d k}+2e^{-\tau_d k}\cos\beta k)(1-e^{-2\tau_d k})}, \tag{9}$$

which can be further approximated by

$$F^{(k)}_{y\to x} \approx \frac{1}{C}e^{-2\tau_d k}\sin^2\beta k, \tag{10}$$

which is Eq. (21) in the main text.

1.2 APPENDIX B

1.2.1 Spectral matrix $\mathbf{S}^{(\tau)}(\omega)$ as $\tau \to 0$ The covariance matrix $\mathbf{G}(n\tau)$ for $X_{n\tau}$, $Y_{n\tau}$ is a sampling of covariance matrix $\mathbf{G}(s)$ for X_t, Y_t, where s is a real value. $\mathbf{G}(s)$ is defined as

$$\mathbf{G}(s) = \begin{bmatrix} \mathrm{cov}(X_t, X_{t-s}) & \mathrm{cov}(X_t, Y_{t-s}) \\ \mathrm{cov}(Y_t, X_{t-s}) & \mathrm{cov}(Y_t, Y_{t-s}) \end{bmatrix}. \tag{11}$$

By the Wiener-Khinchin theorem **Chatfield** (2003), spectral matrix $\mathbf{S}^{(\tau)}(\omega)$ is the Fourier transform of covariance matrix $\mathbf{G}(n\tau)$, that is, $\mathbf{S}^{(\tau)}(\omega) = \sum_{n=-\infty}^{+\infty}\mathbf{G}(n\tau)e^{-in\omega}$. The relation between real frequency f for continuous-time processes and ω in the discrete time is $\omega = 2\pi\tau f$. Then,

$$\mathbf{S}^{(\tau)}(\omega) = \sum_{n=-\infty}^{+\infty}\mathbf{G}(n\tau)e^{-\mathrm{i}n\tau 2\pi f}. \tag{12}$$

By fixing the frequency f and taking the limit of $\tau \to 0$, replacing the summation in Eq. (12) by integration, we have

$$\tau\mathbf{S}^{(\tau)}(\omega) \to \int_{-\infty}^{+\infty}\mathbf{G}(s)e^{-\mathrm{i}s2\pi f}\mathrm{d}s \tag{13}$$

as $\tau \to 0$. Defining

$$\mathbf{P}(f) = \int_{-\infty}^{+\infty}\mathbf{G}(s)e^{-\mathrm{i}s2\pi f}\mathrm{d}s, \tag{14}$$

which is the power spectral density **Rieke et al.** (1999) of continuous process X_t, Y_t, *i.e.*, $\tau\mathbf{S}^{(\tau)}(\omega) \to \mathbf{P}(f)$ as $\tau \to 0$. Rewrite this equation in terms of all the components of the matrix, we obtain

$$\tau\begin{bmatrix} S^{(\tau)}_{xx}(\omega) & S^{(\tau)}_{xy}(\omega) \\ S^{(\tau)}_{yx}(\omega) & S^{(\tau)}_{yy}(\omega) \end{bmatrix} \to \begin{bmatrix} P_{xx}(f) & P_{xy}(f) \\ P_{yx}(f) & P_{yy}(f) \end{bmatrix} \tag{15}$$

as $\tau \to 0$, which is the limiting behavior of spectrum matrix $\mathbf{S}^{(\tau)}(\omega)$ as sampling interval length τ approaches 0.

1.2.2 GC as $\tau \to 0$ For spectral matrix $\mathbf{S}^{(\tau)}(\omega)$, we have the following factorization **Wilson** (1972)

$$\mathbf{S}^{(\tau)}(\omega) = \mathbf{A}^{(\tau)}(e^{\mathrm{i}\omega})\mathbf{A}^{(\tau)}(e^{\mathrm{i}\omega})^*, \tag{16}$$

where $*$ denotes matrix adjoint. The factorization is unique if $\mathbf{A}^{(\tau)}(z)$ and $\mathbf{A}^{(\tau)}(z)^{-1}$ are analytic inside the unit disk and $\mathbf{A}^{(\tau)}(0)$ is real, upper triangular with positive diagonal coefficients **Wilson** (1972). Set $\mathbf{\Sigma}^{(\tau)} = \mathbf{A}^{(\tau)}(0)\mathbf{A}^{(\tau)}(0)^*$, $\mathbf{H}^{(\tau)}(\omega) = \mathbf{A}^{(\tau)}(e^{\mathrm{i}\omega})\mathbf{A}^{(\tau)}(0)^{-1}$, then $\mathbf{S}^{(\tau)}(\omega)$ can be decomposed as

$$\mathbf{S}^{(\tau)}(\omega) = \mathbf{H}^{(\tau)}(\omega)\mathbf{\Sigma}^{(\tau)}\mathbf{H}^{(\tau)}(\omega)^*, \tag{17}$$

where $\mathbf{H}^{(\tau)}(\omega) = \begin{bmatrix} H_{xx}^{(\tau)}(\omega) & H_{xy}^{(\tau)}(\omega) \\ H_{yx}^{(\tau)}(\omega) & H_{yy}^{(\tau)}(\omega) \end{bmatrix}$. By the mean value property of an analytic function, one has $\frac{1}{2\pi}\int_{-\pi}^{\pi}\mathbf{H}^{(\tau)}(\omega)\mathrm{d}\omega = \mathbf{I}$ ($\mathbf{I}$ is the identity matrix). The relation between real frequency f for continuous-time processes and ω in the discrete time is $\omega = 2\pi\tau f$. Then, we obtain $\int_{-\frac{1}{2\tau}}^{\frac{1}{2\tau}} \tau\mathbf{H}^{(\tau)}(2\pi\tau f)\mathrm{d}f = \mathbf{I}$, which implies that $H_{xx}^{(\tau)}$, $H_{xy}^{(\tau)}$ and $H_{yy}^{(\tau)}$ are scaled as $\frac{1}{\tau}$ as $\tau \to 0$ as confirmed in Supplementary Fig. 1 for time series obtained for the neuronal network reconstruction. Combining the scaling of $\mathbf{S}^{(\tau)}$ and $\mathbf{H}^{(\tau)}$, one can see that $\mathbf{\Sigma}^{(\tau)}$ is scaled as τ as $\tau \to 0$. This scaling is confirmed in Supplementary Fig. 1a.

Supplementary Figures 1b and c display the convergence properties of H's, in which we verify that $\tau\mathbf{H}^{(\tau)}$ converges to a limit as the sampling interval length τ approaches 0.

Defining $\hat{\mathbf{H}}(f) = \lim_{\tau\to 0}\tau\mathbf{H}^{(\tau)}(2\pi\tau f)$, $\hat{\mathbf{\Sigma}} = \lim_{\tau\to 0}\frac{1}{\tau}\mathbf{\Sigma}^{(\tau)}$, we can show that $\mathbf{P}(f)$ can be factorized as

$$\mathbf{P}(f) = \hat{\mathbf{H}}(f)\hat{\mathbf{\Sigma}}\hat{\mathbf{H}}(f)^*, \tag{18}$$

where $\int_{-\infty}^{+\infty}\hat{\mathbf{H}}(f)\mathrm{d}f = \mathbf{I}$. Using the components in the factorization (17), the sampled Granger causalities using the frequency domain decomposition **Geweke** (1982); **Ding et al.** (2006), as $\tau \to 0$, become Eqs. (26) (27) and (28) in the main text.

Note that $F_{x,y}^{(\tau)}$ is the sum of its positive components $F_{x\to y}^{(\tau)}$, $F_{y\to x}^{(\tau)}$ and $F_{x\cdot y}^{(\tau)}$, therefore $F_{x,y}^{(\tau)}$ is larger than any of its components. If $\int_{-\infty}^{+\infty}\ln\left[1 - C(f)\right]\mathrm{d}f$ is finite, then we can easily show that $\frac{1}{\tau}F_{x,y}^{(\tau)}$, $\frac{1}{\tau}F_{x\to y}^{(\tau)}$, $\frac{1}{\tau}F_{y\to x}^{(\tau)}$ and $\frac{1}{\tau}F_{x\cdot y}^{(\tau)}$ all approach finite values in the limit of $\tau \to 0$. As mentioned in the main text, these limits are related to intrinsic properties of continuous time processes. Therefore, the Granger causality is linearly proportional to the sampling interval length τ for small τ.

1.3 APPENDIX C

From our observation, the GC values are very small as sampling interval length $\tau \to 0$. Under this condition, we should consider the estimator bias of GC in order to precisely recover the limit GC sampling structure as $\tau \to 0$ through numerical approach. From Ref. **Geweke** (1982), for bivariate time series X_t, Y_t, if the true GC value $F_{x\to y}$, $F_{y\to x}$, $F_{x\cdot y}$ and $F_{x,y}$ are 0, then, $n\hat{F}_{x\to y} \sim \chi^2(p)$, $n\hat{F}_{y\to x} \sim \chi^2(p)$, $n\hat{F}_{x\cdot y} \sim \chi^2(1)$, $n\hat{F}_{x,y} \sim \chi^2(2p+1)$, where p is the regression order, n is the length of time series, the caret symbol denotes the sample estimate. If $F_{x\to y}$, $F_{y\to x}$, $F_{x\cdot y}$ and $F_{x,y}$ are positive, then $n\hat{F}_{x\to y} \sim \chi'^2(p, nF_{x\to y})$, $n\hat{F}_{y\to x} \sim \chi'^2(p, nF_{y\to x})$, $n\hat{F}_{x\cdot y} \sim \chi'^2(1, nF_{x\cdot y})$, $n\hat{F}_{x,y} \sim \chi'^2(2p+1, nF_{x,y})$, where χ' is the noncentral chi-square distribution. Therefore, the estimate biases of GC values are $\Delta F_{x\to y} = \mathrm{E}(\hat{F}_{x\to y} - F_{x\to y}) = \frac{p}{n}$, $\Delta F_{y\to x} = \mathrm{E}(\hat{F}_{y\to x} - F_{y\to x}) = \frac{p}{n}$,

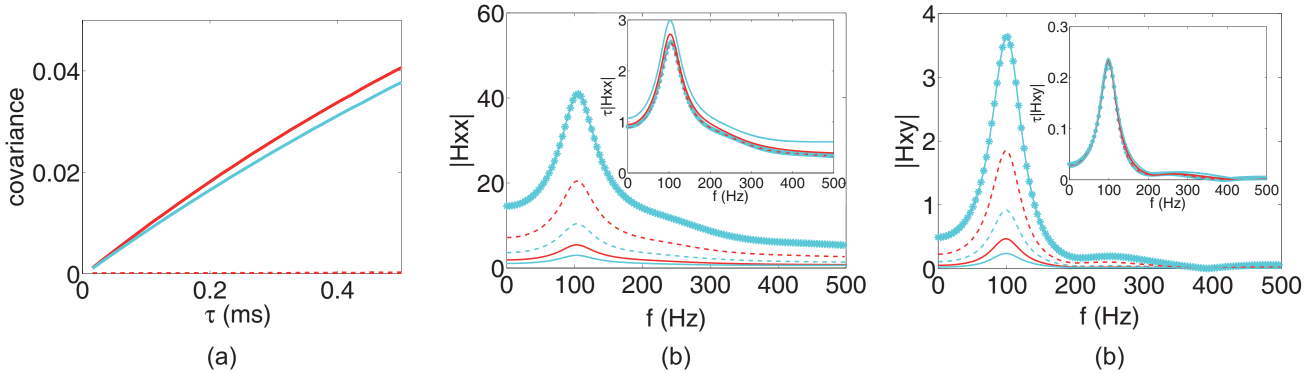

Supplementary Figure 1.(a) The covariance $\Sigma_2^{(\tau)}$ (red), $\Gamma_2^{(\tau)}$ (cyan) and $\Upsilon_2^{(\tau)}$ (dash red) as a function of the sampling interval τ. (b) $\left|H_{xx}^{(\tau)}\right|$. Inset: $\tau\left|H_{xx}^{(\tau)}\right|$. (c) $\left|H_{xy}^{(\tau)}\right|$. Inset: $\tau\left|H_{xy}^{(\tau)}\right|$. Sampling interval lengths are $0.0625\,\mathrm{ms}$ (cyan star), $0.125\,\mathrm{ms}$ (dash red), $0.25\,\mathrm{ms}$ (dash cyan), $0.5\,\mathrm{ms}$ (red), $1\,\mathrm{ms}$ (cyan) respectively. The time series are generated by a two-neuron I&F network with parameters $\nu = 1\,\mathrm{ms}^{-1}$, $\lambda = 0.0177$, $s_{xy} = 0$, $s_{yx} = 0.02$.

$\Delta F_{x\cdot y} = \mathrm{E}(\hat{F}_{x\cdot y} - F_{x\cdot y}) = \frac{1}{n}$, $\Delta F_{x,y} = \mathrm{E}(\hat{F}_{x,y} - F_{x,y}) = \frac{2p+1}{n}$ regardless of whether the true value of GC vanishes or not. We can faithfully recover the limit GC sampling structure by subtracting the estimator biases from the numerical estimators of GC values. Note that this approach may lead to slightly negative numerical GC values due to statistical fluctuations if the theoretical value of GC vanishes originally.

SUPPLEMENTARY FIGURES

REFERENCES

Chatfield, C. (2003), The analysis of time series: an introduction, volume 59 (Chapman & Hall/CRC)

Ding, M., Chen, Y., and Bressler, S. L. (2006), Granger causality: Basic theory and application to neuroscience, in B. Schelter, t. Winterhalder, and J. Timmer, eds., Handbook of Time Series Analysis (Wiley-VCH Verlag GmbH & Co. KGaA), 437–460

Geweke, J. (1982), Measurement of linear dependence and feedback between multiple time series, *Journal of the American Statistical Association*, 77, 378, 304–313

Rieke, F., Warland, D., van Steveninck, R. d. R., and Bialek, W. (1999), Spikes: exploring the neural code (MIT press)

Wilson, G. T. (1972), The factorization of matricial spectral densities, *SIAM Journal on Applied Mathematics*, 23, 4, 420–426

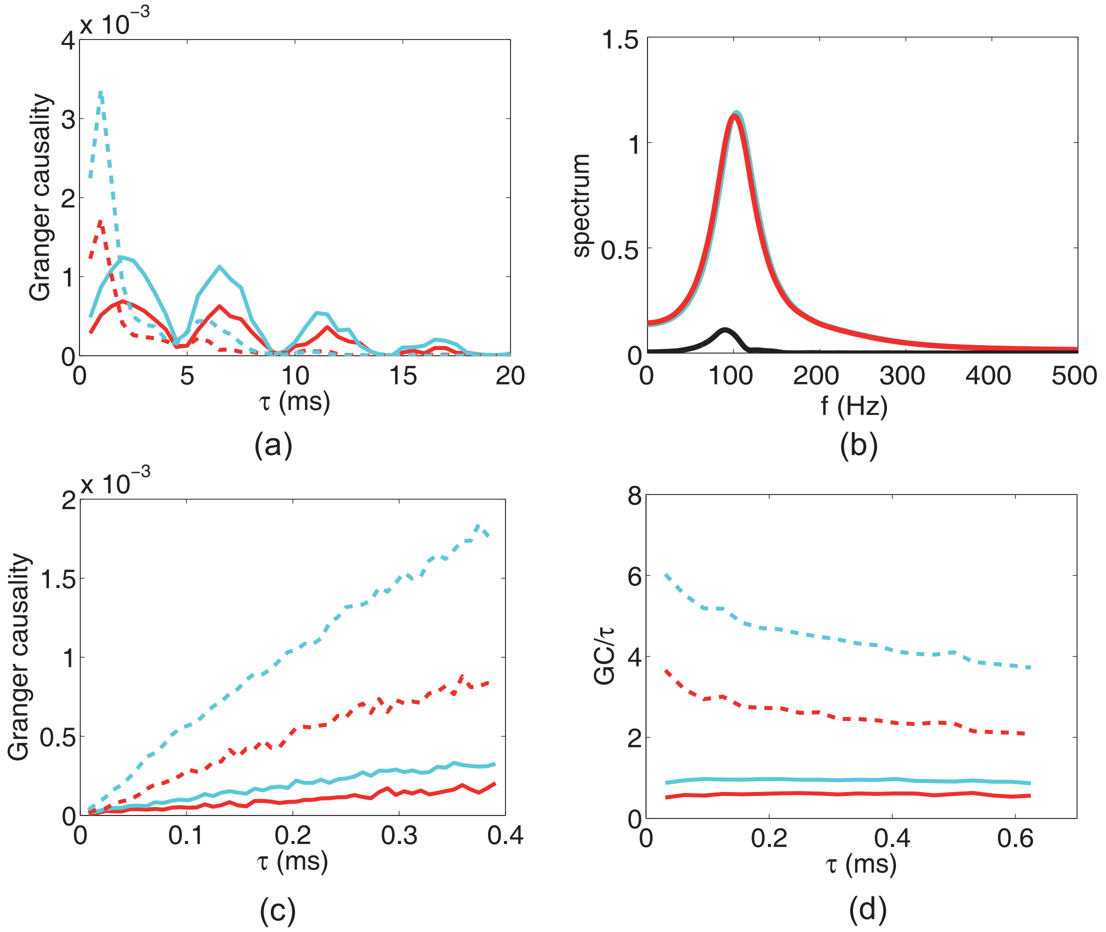

Supplementary Figure 2. The GC sampling structure for the bidirectional two-neuron network. Parameters of the network are $\nu = 1ms^{-1}$, $\lambda = 0.0177$, $s_{xy} = 0.015$ and $s_{yx} = 0.02$. (a) GC vs. sampling interval length: $F_{x\to y}$ (red), $F_{y\to x}$ (cyan) obtained from voltage time series and $F_{x\to y}$ (red dash), $F_{y\to x}$ (cyan dash) obtained from spike train time series with sampling interval τ. (b) The corresponding spectra of the voltage time series for (a): S_{xx} (cyan), S_{yy} (red), $|S_{xy}|$ (black). (c) The GC sampling structure as sampling interval length tends to zero. $F_{x\to y}$ (red), $F_{y\to x}$ (cyan) obtained from voltage time series and $F_{x\to y}$ (red dash), $F_{y\to x}$ (cyan dash) obtained from spike train time series with sampling interval length τ. (d) $\frac{1}{\tau}F^{(\tau)}_{x\to y}$ (red), $\frac{1}{\tau}F^{(\tau)}_{y\to x}$ (cyan) for voltage time series and $\frac{1}{\tau}F^{(\tau)}_{x\to y}$ (red dash), $\frac{1}{\tau}F^{(\tau)}_{y\to x}$ (cyan dash) for spike train time series vs. sampling interval length τ. Note that we have subtracted the estimation bias of GC from the estimate of GC values for (c) and (d). The procedure of removing biases is described in Appendix C.
